# Environmental conditions affect the nutritive value and alkaloid profiles of *Lupinus* forage: Opportunities and threats for sustainable ruminant systems

**DOI:** 10.1016/j.heliyon.2024.e28790

**Published:** 2024-03-27

**Authors:** Ana R.J. Cabrita, Inês M. Valente, André Monteiro, Carla Sousa, Carla Miranda, Agostinho Almeida, Paulo P. Cortez, Carlos Castro, Margarida R.G. Maia, Henrique Trindade, António J.M. Fonseca

**Affiliations:** a REQUIMTE, LAQV, ICBAS, School of Medicine and Biomedical Sciences, University of Porto, Rua Jorge Viterbo Ferreira, 228, 4050-313, Porto, Portugal; b REQUIMTE, LAQV, Department of Chemistry and Biochemistry, Faculty of Sciences, University of Porto, Rua do Campo Alegre 687, 4169-007, Porto, Portugal; c Center for the Research and Technology Agro-Environmental and Biological Sciences (CITAB), Universidade de Trás-os-Montes e Alto Douro, Quinta de Prados, 5000-801, Vila Real, Portugal; d REQUIMTE, LAQV, Department of Chemical Sciences, Laboratory of Applied Chemistry, Faculty of Pharmacy, University of Porto, Rua Jorge Viterbo Ferreira, 228, 4050-313, Porto, Portugal; e CECA/ICETA, ICBAS, School of Medicine and Biomedical Sciences, University of Porto, Rua Jorge Viterbo Ferreira, 228, 4050-313, Porto, Portugal

**Keywords:** Alkaloids, Forage, *Lupinus*, Nutritive value

## Abstract

The identification of crops that simultaneously contribute to the global protein supply and mitigate the effects of climate change is an urgent matter. Lupins are well adapted to nutrient-poor or contaminated soils, tolerate various abiotic stresses, and present relevant traits for acting as ecosystem engineers. Lupins are best studied for their seeds, but their full foraging potential needs further evaluation. This study evaluated the effects of location and sowing date on forage production, proximate composition, and the detailed mineral and alkaloid profiles of three species of *Lupinus* (*L. albus* cv. Estoril, *L. angustifolius* cv. Tango, and *L. luteus* cv. Cardiga). Sowing date and location and their interaction with the plant species significantly affected the vast majority of measured parameters, emphasizing the effects of climate and soil conditions on these crops. The relatively high crude protein and *in vitro* digestibility support the potential of the lupin species studied as sustainable forage protein sources in diets for ruminant animals. The content of individual essential macro and trace elements was below the maximum tolerable levels for cattle and sheep. Lupanine, smipine, and sparteine were the most abundant quinolizidine alkaloids in *L. albus* cv. Estoril, lupanine, and sparteine in *L. angustifolius* cv. Tango, and lupinine, gramine, ammodendrine, and sparteine in *L. luteus* cv. Cardiga. Based on the maximum tolerable levels of total quinolizidine alkaloid intake, the dietary inclusion of forages of *L. albus* cv. Estoril and *L*. *angustifolius* cv. Tango does not pose a risk to the animals, but the high alkaloid content of *L. luteus* cv. Cardiga may compromise its utilization at high levels in the diet. Overall, the results reveal a high potential for lupins as protein forage sources well adapted to temperate regions and soils with lower fertility, with a relevant impact on livestock sustainability in a climate change era.

## 1 Introduction

The imbalance between the supply and demand of plant proteins and climate change has been the subject of debate in Europe and worldwide [1,2]. The imbalance makes it urgent to identify crops that simultaneously contribute to the global protein supply and mitigate the threats of climate change. In this context, legume production plays an important role as a source of dietary protein [3] and because of the multifaceted impact it has on ecosystem services. The ability of legumes to fix atmospheric nitrogen (N_2_) reduces crop inorganic fertilizer needs and related costs and contributes to increasing soil fertility and the production of subsequent crops in rotation systems [4]. Additionally, intercropping and rotating legumes with nonlegume crops such as cereals have been widely documented to reduce the occurrence of plant diseases, improve access to soil essential elements (*e.g.*, phosphorous, P), and reduce greenhouse gas emissions [[5], [6], [7], [8]]. Legumes also play an important role in carbon sequestration and thus in reducing carbon footprints, mainly due to their deep root systems, leaf fall, N_2_ fixation, and carbon-rich root exudates [9]. In the context of climate change, legumes might be in an advantageous position to increase production through various strategies (*e.g.*, increased rate of photosynthesis, increased content of soluble sugars and growth-promoting hormones, and increased N_2_ fixation) obtained from their C3 pathways, favored by elevated carbon dioxide levels [10]. Legumes also perform well in less fertile soils and withstand abiotic stresses [11].

Among legumes, lupins are considered more tolerant to various abiotic stresses, showing high resilience in nutrient-poor or contaminated soils [12]. The ability of lupins to symbiotically fix N_2_ and their potential to mobilize sparingly available nutrients, particularly P and micronutrients, for themselves and for interplanted or subsequent crops constitute relevant traits for acting as “ecosystem engineers” [6]. Additionally, as lupins are capable of taking up moderate amounts of heavy metals (*e.g.*, cadmium, Cd; lead, Pb; and mercury, Hg) from the soil, they may play an important role in phytoremediation [13]. Although best studied for their seeds, lupins are also cultivated to be used as green manure or as a whole crop for animal feeding, either directly grazed or harvested and conserved as hay or silage [14,15]. However, as the low dry matter (DM) and soluble sugar content of lupins make the ensiling process difficult, the bi-crop approach should be considered [16,17].

Lupins are known to contain toxic alkaloids, especially indole and quinolizidine alkaloids, synthesized as part of a defense strategy against herbivores and pathogenic microorganisms, having allelopathic functions and probably constituting N reserves [18]. Although a high alkaloid content may be considered a preferable trait in green manure cultivars due to its decontaminating effect on the soil, thereby decreasing the negative impacts of diseases and pests on subsequent crops [19], recent concerns have arisen due to the leaching of alkaloids into agricultural drainage water, contributing to water contamination [20]. The use of lupins in animal feeding may be limited by their alkaloid content, since several negative effects on animal health have been reported, including acute and chronic symptoms [21,22], and their bitter taste can negatively affect zootechnical performance due to palatability issues [23]. Although differences in microbiota composition in cows with different tolerances to fescue toxicosis [24] suggest that the microbiome of ruminant animals partially metabolizes alkaloids into nontoxic compounds, some evidence suggests the transfer of alkaloids to meat and milk [25], potentially undermining food safety. Conversely, alkaloids have been assigned several therapeutic properties, such as antioxidant, anti-inflammatory, and vasodilatory effects [25], but their benefits for livestock remain poorly studied. Interestingly, although the type and level of alkaloids are known to depend on the plant genotype, presence of pathogens, environmental effects, and soil characteristics [26], no data are available for quinolizidine alkaloids in whole crops or ensiled lupins.

In fact, the characterization of the genetic resources conserved by GenBank is generally limited in what concerns lupins, emphasizing the need to better characterize the genetic resources using modern genetic and metabolomics techniques. Czepiel et al. [27], assessing the breeding progress toward reducing the total alkaloid content of yellow lupin cultivars from the Polish *Lupinus* Collection, concluded that the results obtained highlighted the importance of characterizing and conserving genetic resources with metabolomics-assisted breeding to hasten the selection of improved breeding materials. Additionally, Zafeiriou et al. [28] evaluated the genetic diversity of white lupin resources in Greece using morphological and molecular markers and found a high degree of landrace accession polymorphism, suggesting the importance of the development of molecular markers for modern breeding. The use of molecular markers associated with the content of quinolizidine alkaloids in narrow-leafed lupins has been previously described by Li et al. [29]. Moreover, the identification and manipulation of genes involved in the biosynthesis of quinolizidine alkaloids will enable precision breeding of low-alkaloid and high-nutritive lupins [30].

The study presented here focused on three species of *Lupinus* (*L*. *albus* cv. Estoril, *L. angustifolius* cv. Tango, and *L. luteus* cv. Cardiga) well adapted to the Mediterranean region. Forages were harvested from the field experiment presented by Monteiro et al. [31] with the aim of understanding the effects of location and sowing date on proximate composition and detailed mineral and alkaloid profiles. The results are expected to contribute to the improvement of temperate agricultural ecosystems and to drive geneticists to improve local varieties, thus contributing to improve the rural economy and reducing desertification.

## 2 Material and methods

### 2.1 Location and edaphoclimatic characteristics

The experiments were conducted between September 2018 and June 2019, simultaneously in Mirandela (41.511896, −7.161595) and Vila Real (41.284747, −7.738875) and in northeastern Portugal. The soil in Mirandela was a Eutric Fluvisol of unconsolidated material >1 m deep (IUSS Working Group WRB, 2015), and in Vila Real it was a shallow Dystric Cambisol obtained from schist. During the field experiment, the maximum and minimum average temperatures were higher than the historical average (1971–2010) for both locations. Conversely, the average precipitation was lower, particularly in Vila Real, with the exception of the months of November 2018 and April 2019, which recorded high rainfall. The main properties of the soils and climatic conditions during the field experiment are presented as supplementary material (Table S1 and Fig. S1).

### 2.2 Experimental design and treatments

The trial was conducted in a randomized complete block design with four replications and three factors: location (Mirandela and Vila Real), sowing date (D1, September 18, 2018; D2, October 11, 2018; D3, November 8, 2018; D4, November 28, 2018), and *Lupinus* species (*L. albus* cv. Estoril, *L. angustifolius* cv. Tango, and *L. luteus* cv. Cardiga), totaling 48 plots (each with a rectangular section of 2.5 × 4.0 m, resulting in 10 m^2^) in each location.

Fifteen days before the first sowing, the soils were mobilized by tillage followed by cross-scarification, reaching a mobilized depth of approximately 20 cm. The sowing density was set at 100 kg ha^−1^ for *L. albus* cv. Estoril, 80 kg ha^−1^ for *L. angustifolius* cv. Tango, and 60 kg ha^−1^ for *L. luteus* cv. Cardiga. Sowing was conducted manually at a depth of 3–4 cm in rows 30 cm apart. The distance from seed to seed in the row was approximately 10 cm for *L. albus* cv. Estoril and 5 cm for *L. angustifolius* cv. Tango and *L*. *luteus* cv. Cardiga. No other agricultural operation was conducted until the harvest, with the crops being exclusively rainfed. Regardless of the sowing date, all plants were harvested on May 21 in the state of pods with doughy grain. In both locations, whole plants from 0.6 m^2^ of each plot were harvested, eliminating the two extreme lines to avoid border effects, weighed, and dried at 65 °C for 48 h in a forced air oven for further analyses.

### 2.3 Chemical analyses

#### 2.3.1 Proximate composition

Forage samples were milled at a 1-mm screen, and proximate composition was determined in duplicate according to official methods [32]. Samples were analyzed for DM (ID 934.01), ash (ID 942.05), ether extract (EE; ID 920.39), and Kjeldahl N (ID 954.01). Crude protein (CP) was calculated as Kjeldahl N × 6.25. Neutral detergent fiber (NDF; without sodium sulfite), acid detergent fiber (ADF) and acid detergent lignin (ADL) were determined according to Robertson et al. [33] and Van Soest et al. [34].

#### 2.3.2 Mineral profile

Macro and trace elements were analyzed as described by Cabrita et al. [35]. Afterward, forage samples were mineralized using a Milestone (Sorisole, Italy) MLS 1200 Mega high-performance microwave digestion unit, and sample solutions were analyzed using inductively coupled plasma-mass spectrometry (ICP-MS; iCAP Q ICP-MS instrument, Thermo Fisher Scientific, Waltham, MA, USA) and flame atomic absorption spectrometry (FAAS; AAnalyst 200 FAAS instrument, PerkinElmer, Shelton, CT, USA). Internal standards and tuning solutions for ICP-MS analysis were prepared by appropriate dilution of the following commercial solutions: periodic table mix 3 for ICP-MS (TraceCERT®, Sigma-Aldrich, Buchs, Switzerland) containing 10 mg L^−1^ of 16 elements (Sc, Y, La, Ce, Pr, Nd, Sm, Eu, Gd, Tb, Dy, Ho, Er, Tm, Yb, and Lu in 5% HNO_3_) and a custom solution (obtained from SCP Science, Baie D'Urfé, QC, Canada) with 1 mg L^−1^ of barium (Ba), Bi, Ce, cobalt (Co), In, lithium (Li) and U in 5% HNO_3_ + 0.5% HCl. Calibration standards to run FAAS analysis were prepared from 1000 mg L^−1^ single-element standard stock solutions (Fluka, Buchs, Switzerland) by appropriate dilution with HNO_3_ 0.2% (v/v). Analyses were made in triplicate.

#### 2.3.3 Alkaloid composition

Alkaloids were extracted in duplicate according to the procedure of Magalhães et al. [36]. Briefly, extraction was performed with 5% trichloroacetic acid under constant stirring for 30 min, with subsequent alkalinization of the supernatant with sodium hydroxide. The aqueous extract was purified by liquid–liquid extraction with dichloromethane, and the organic solvent evaporated. The alkaloid-rich residue was stored, protected from light, at −20 °C, until analysis. The extracts were dissolved in dichloromethane and filtered with a 0.45-μm regenerated cellulose syringe filter. The chromatographic analysis was performed using a Thermo Scientific Trace 1300, ISQ Single Quadrupole MS GC–MS system equipped with a TraceGOLD TG-5MS column (30 m × 0.25 mm × 0.25 μm; Thermo Scientific) according to the protocol described by Maia et al. [37].

### 2.4 *In vitro* digestibility

*In vitro* digestibility was determined using a Daisy^II^ incubator unit (Ankom Technology, Macedon, NY, USA). Forage samples (250 mg) were heat-sealed into F57 fiber filter bags (25 μm pore size; Ankom Technology), previously rinsed with acetone, and air-dried using an impulse sealer (Type AIE-200, 50/60 HZ, Ankom Technology). Filter bags containing each of the 96 samples (3 *Lupinus* species × 2 locations × 4 sowing dates × 4 replicates), along with one forage standard (haylage) and blanks, were incubated for 48 h with rumen inocula.

Three lactating Holstein cows equipped with a ruminal cannula (10 cm in diameter; Bar Diamond Inc., Parma, ID, USA) served as donors for the rumen fluid used in the inoculum. The cows were housed at the Vairão Agricultural Campus of the School of Medicine and Biomedical Sciences, University of Porto (ICBAS-UP, Vila do Conde, Portugal), following EU good animal practices for the protection of animals used for scientific purposes (Directive, 2010/63/EU). The care and maintenance procedures for donor cows were approved by the ICBAS-UP Animal Ethics Committee, licensed by the Portuguese General Directorate for Food and Veterinary (permit #0421/000/000/2021), and conducted by trained scientists (FELASA category C). Cows were fed a diet composed (on a DM basis) of maize silage (51.5%), haylage (2.4%), chopped barley straw (10.6%), compound feed (32.0%), and soybean meal (3.5%), which contained 14.1% CP, 43.0% NDF, and 25.4% starch. The daily ration was offered as 2 equal meals at 8:00 a.m. and 6:15 p.m. Cows had free access to fresh drinking water and mineral salt blocks.

Ruminal contents were collected before the morning feed, filtered through four layers of gauze, and combined with equal contributions from the three cows. A total of 400 mL of rumen fluid were added to each incubation jar containing buffer [1 strained rumen fluid:4 Kansas State buffer; [38] and sample bags, and the headspace was purged with carbon dioxide. Following incubation, bags containing residues of forages and blanks were digested in a neutral detergent solution [33] for 1 h and then dried overnight in an air-convection oven at 45 °C. After weighing, residues were incinerated in a muffle furnace at 500 °C for 6 h for ash correction. *In vitro* DM and organic matter (OM) digestibility was calculated as the difference between the incubated DM or OM and the undigested residue, correcting for blank residues. A total of three incubation runs were performed on independent days.

### 2.5 Statistical analyses

Statistical analyses were performed using the General Linear Model and Linear Regression Model procedures of the SAS software program (2022; Academic version, SAS Institute Inc., Cary, NC, USA). The statistical model included the fixed effect of *Lupinus* species, location, sowing date, all double and triple interactions, and the random residual error. As the triple interaction species × location × sowing date was never significant, it was removed from the model. Significance was assumed for *p* < 0.05, and multiple comparisons of means were performed using Tukey's posthoc test. Plots were constructed in R software (version 4.3.2) using “ggplot2” and “ggbreak” [39] packages.

The effects of the environmental factors (average mean, maximum and minimum temperatures, and total rainfall during the cultivation period for each location) on the productivity, chemical composition, and alkaloid content of *Lupinus* forages were performed in R software. Principal component analysis (PCA) was used to characterize the correlation pattern between the environmental variables and the genotypes through the responses of productivity (DM and protein), proximate chemical composition, and total alkaloid content. The analysis was done using the packages “nFactors” (version 2.4.1.1), and the results were displayed after Varimax rotation in a distance biplot performed using the packages “FactoMineR” (version 2.9) and “factoextra” (version 1.0.7). To study the environmental variables with an impact on the productivity and total alkaloid content of *Lupinus* forages, a ReDundancy Analysis (RDA) was performed using the “vegan” package (version 2.6–4) in R.

## 3 Results

### 3.1 Forage production, proximate composition, and digestibility

Table 1 presents the effects of species, sowing location and date, and their interactions on proximate composition, *in vitro* digestibility, and estimated metabolizable energy content (treatment means ± standard deviation are presented as supplementary material, Table S2).

**Table 1:** Effect of species, sowing date, and location on the chemical composition (g 100 g^−1^ DM), DM (DMD; g 100 g^−1^), and organic matter (OMD; g 100 g^−1^ DM) digestibility and estimated metabolizable energy (ME; MJ g 1000 g^−1^ DM) in the studied *Lupinus* species.

	DM (%)	Ash	CP	EE	NDF	ADF	ADL	NSC	DMD	OMD	ME
Species											
*Lupinus albus* cv. Estoril	18.7^b^	4.62^a^	14.8^a^	0.852^a^	44.3^a^	34.8	6.26	34.6^c^	71.4^b^	71.2^b^	11.7^b^
*Lupinus angustifolius* cv. Tango	19.3^b^	7.07^c^	15.3^a^	1.11^b^	45.1^b^	35.3	6.30	31.5^b^	69.2^a^	68.1^a^	11.2^a^
*Lupinus luteus* cv. Cardiga	15.8^a^	6.50^b^	16.5^b^	1.66^c^	46.0^b^	35.9	6.32	29.3^a^	70.1^ab^	69.0^ab^	11.5^ab^
Sowing date											
D1	16.1^a^	6.21^b^	15.6	1.23	46.3^b^	36.7^c^	6.72^b^	30.0^a^	68.5^a^	67.2^a^	11.2^a^
D2	15.7^a^	6.18^b^	15.7	1.26	46.3^b^	36.2^c^	6.52^b^	30.6^a^	68.6^a^	67.8^a^	11.2^a^
D3	21.3^c^	5.79^a^	15.2	1.12	44.6^a^	34.9^b^	6.12^a^	33.3^b^	71.4^b^	70.6^b^	11.6^b^
D4	18.5^b^	6.07^b^	15.6	1.23	43.3^a^	33.4^a^	5.83^a^	33.2^b^	72.6^b^	72.1^b^	11.8^b^
Location											
Mirandela	18.9	6.28	18.0	1.20	44.2	34.8	6.02	29.7	72.4	71.5	11.9
Vila Real	17.0	5.84	13.0	1.21	46.1	35.8	6.57	33.8	68.1	67.4	10.9
Statistics											
*p*-Values											
Species	<0.001	<0.001	<0.001	<0.001	0.044	0.107	0.930	<0.001	0.004	0.004	0.001
Sowing date	0.007	0.004	0.555	0.239	<0.001	0.013	<0.001	<0.001	<0.001	<0.001	<0.001
Location	<0.001	<0.001	<0.001	0.896	0.001	<0.001	<0.001	<0.001	<0.001	<0.001	<0.001
Species × sowing date	0.615	0.114	0.931	0.236	0.002	<0.001	0.308	0.005	0.484	0.642	0.728
Species × location	0.173	<0.001	<0.001	0.010	0.007	0.004	0.481	<0.001	0.074	0.187	0.740
Sowing date × location	<0.001	0.121	0.082	0.112	0.018	0.028	0.006	0.002	0.162	0.096	0.385
RSD	3.67	0.481	1.57	0.285	3.09	2.21	0.683	3.11	2.98	3.33	0.569
R^2^	0.526	0.882	0.792	0.669	0.457	0.505	0.426	0.672	0.575	0.553	0.596
Adjusted R^2^	0.421	0.856	0.746	0.597	0.339	0.397	0.301	0.601	0.482	0.455	0.508

CP, crude protein; EE, ether extract; NDF, neutral detergent fiber; ADF, acid detergent fiber; ADL, acid detergent lignin; NSC, nonstructural carbohydrates. NSC is calculated as DM − ash − CP − EE − NDF. Metabolizable energy is estimated according to Givens et al. [71]: ME (MJ g 1000 g^−1^ DM) = 0.37 + 0.0142 OMD (g 1000 g^−1^) + 0.0077 CP (g 1000 g^−1^ DM). RSD, residual standard deviation; R^2^, coefficient of determination. ^a,b^Means within a column with different superscript letters are significantly different (*p* < 0.05).

**Fig. 1:**
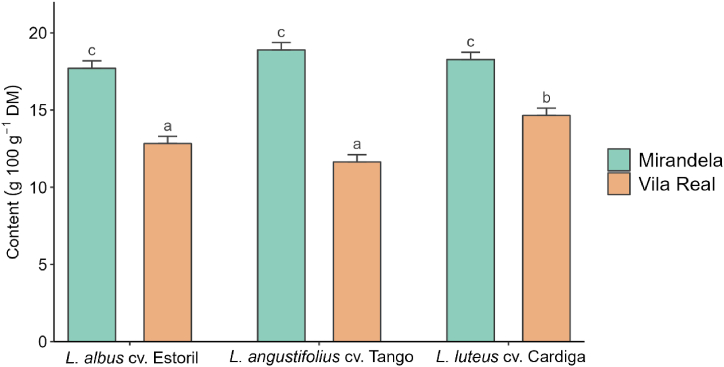
Effect of interaction between species and location on crude protein (CP) content in different *Lupinus* species. ^a,b,c^Means with different superscript letters are significantly different (*p* < 0.05).

*L*. *luteus* cv. Cardiga presented the lowest DM content, with no differences between Estoril and Tango cultivars (*p* < 0.001). For the first, second, and fourth sowing dates, the DM content was similar between locations, but on the third date, the DM content was higher in Mirandela than in Vila Real (*p* < 0.001; Fig. S2A). Ash content was the lowest on the third sowing date (5.8%, DM basis), with the other dates not differing from each other. Ash content was lower in Vila Real than in Mirandela for *L*. *luteus* cv. Cardiga, the opposite being observed for *L. angustifolius* cv. Tango, with no differences between locations for *L. albus* cv. Estoril (*p* < 0.001; Fig. S3A). Regardless of the species, CP content was higher in forages from Mirandela, but when sown in Vila Real, CP content was higher for *L. luteus* cv. Cardiga, with this content being similar between *L. albus* cv. Estoril and *L*. *angustifolius* cv. Tango (*p* < 0.001; Fig. 1). The sowing date did not affect CP and EE contents, but EE content was higher for *L. albus* cv. Estoril sown in Vila Real than in Mirandela, with the opposite occurring with *L. angustifolius* cv. Tango (*p* = 0.010; Fig. S3B). The lowest NDF content was observed for *L. albus* cv. Estoril sown on the fourth sowing date (*p* = 0.002; Fig. S4A), and although the NDF content of *L. angustifolius* cv. Tango did not differ between locations, that of *L. luteus* cv. Cardiga was higher when sown in Vila Real (*p* = 0.007; Fig. S3C). Furthermore, the NDF content was higher on the first two dates in Vila Real (*p* = 0.007), with no differences being observed in the remaining sowing dates and locations (Fig. S2B). The lowest ADF content was observed in *L. albus* cv. Estoril sown on the fourth date (*p* < 0.001; Fig. S4B), and higher values were observed in *L. luteus* cv. Cardiga and *L*. *albus* cv. Estoril sown in Vila Real, with the opposite being observed for *L. angustifolius* cv. Tango (*p* = 0.004; Fig. S3D). In addition, the ADF content was higher on the first two sowing dates in Vila Real, with no differences between the other dates and locations (*p* = 0.004; Fig. S2C). The ADL content was significantly affected by the interaction between sowing date and location (*p* = 0.006; Fig. S2D), with ADL content differing between locations only on the second and fourth sowing dates, with the highest values found in Vila Real. The content of nonstructural carbohydrates was generally higher on the third and fourth sowing dates, particularly in Vila Real and for *L. albus* cv. Estoril (Figs. S2E, S3E, and S4C). DM and OM digestibility and estimated metabolizable energy content were higher for *L. albus* cv. Estoril (*p* = 0.004), and regardless of the species, the highest values were observed on the third and fourth dates (*p* < 0.001) and in Mirandela (*p* < 0.001; Table 1).

Forage production was significantly affected by lupin species (Table 2 and Table S3), with the production of *L. angustifolius* cv. Tango being particularly low, almost half of that obtained for *L. albus* cv. Estoril and *L*. *luteus* cv. Cardiga. The lowest productions were observed for the fourth sowing date (*p* < 0.001) and the highest at Mirandela (*p* < 0.001; Table 2).

**Table 2:** Effect of species, sowing date, and location on the production (t dry matter, DM, ha^−1^) of forage DM (PDM), crude protein (PCP), digestible DM (PDMD), digestible organic matter (PDOM), and estimated metabolizable energy (PME; GJ ha^−1^) in the studied *Lupinus* species.

	PDM	PCP	PDMD	PDOM	PME
Species					
*Lupinus albus* cv. Estoril	4.86^b^	0.766^b^	3.46^b^	3.44^b^	56.6^b^
*Lupinus angustifolius* cv. Tango	2.74^a^	0.425^a^	1.89^a^	1.86^a^	30.8^a^
*Lupinus luteus* cv. Cardiga	4.48^b^	0.749^b^	3.14^b^	3.08^b^	51.2^b^
Sowing date					
D1	4.76^b^	0.752^b^	3.30^b^	3.25^b^	53.7^b^
D2	4.64^b^	0.728^b^	3.17^b^	3.14^b^	51.9^b^
D3	4.25^b^	0.683^b^	3.05^b^	3.02^b^	49.7^b^
D4	2.45^a^	0.423^a^	1.79^a^	1.78^a^	29.4^a^
Location					
Mirandela	4.75	0.853	3.43	3.39	56.5
Vila Real	3.30	0.440	2.23	2.20	35.9
Statistics					
*p*-Values					
Species	<0.001	<0.001	<0.001	<0.001	<0.001
Sowing date	<0.001	<0.001	<0.001	<0.001	<0.001
Location	<0.001	<0.001	<0.001	<0.001	<0.001
Species × sowing date	0.397	0.317	0.420	0.417	0.402
Species × location	0.668	0.308	0.629	0.645	0.608
Sowing date × location	0.133	0.207	0.168	0.194	0.195
RSD	2.170	0.331	1.528	1.511	24.68
R^2^	0.422	0.527	0.433	0.432	0.443
Adjusted R^2^	0.294	0.422	0.307	0.306	0.320

Metabolizable energy is estimated according to Givens, Everington [71]: ME (MJ g 1000 g^−1^ DM) = 0.37 + 0.0142 OMD (g 1000 g^−1^) + 0.0077 CP (g 1000 g^−1^ DM). RSD, residual standard deviation; R^2^, coefficient of determination. ^a,b^Means within a column with different superscript letters are significantly different (*p* < 0.05).

### 3.2 Mineral profile

**Fig. 2:**
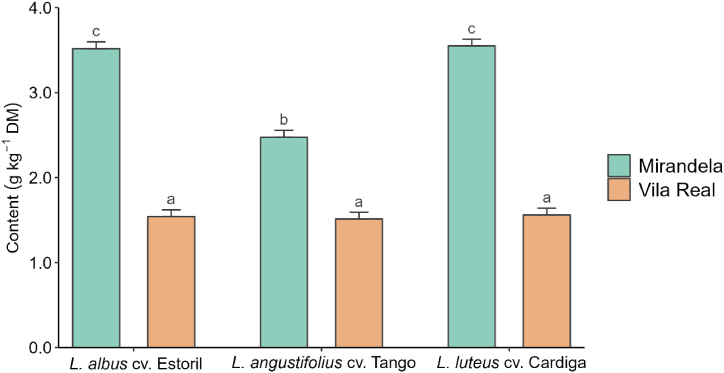
Effect of interaction between species and location on phosphorous (P) content in different *Lupinus* species. ^a,b,c^Means with different superscript letters are significantly different (*p* < 0.05).

Table 3 presents the results regarding the effect of species, sowing date, and location on the content of essential macro and trace elements in forages (treatment means ± standard deviation are presented in Table S4). All essential elements were below the maximum tolerable levels in feeds for cattle and sheep [40]. Calcium (Ca), phosphorus (P), and potassium (K) contents were not affected by the sowing date but were affected by the interaction between species and sowing location. The Ca content was higher when *L. albus* cv. Estoril and *L*. *angustifolius* cv. Tango were sown in Vila Real than in Mirandela, with the opposite occurring for *L. luteus* cv. Cardiga (*p* < 0.001; Fig. S3F). Regardless of the species, the P content was lower in Vila Real than in Mirandela, with the highest values observed for *L. luteus* cv. Cardiga and *L*. *angustifolius* cv. Tango (*p* < 0.001; Fig. 2). These species also showed the highest levels of K when sown in Mirandela, with no differences between locations for *L. albus* cv. Estoril (*p* < 0.001; Fig. S3G). Magnesium (Mg) content was the highest in *L. angustifolius* cv. Tango sown on the first two dates and the lowest in *L. albus* cv. Estoril, regardless of the sowing date (Fig. S4D). Although the Mg content of *L. angustifolius* cv. Tango did not differ between locations, for the other two species, it was higher in plants sown in Mirandela than in Vila Real (Fig. S3H). Sodium (Na) content was affected only by the *Lupinus* species (*p* = 0.013), with the lowest value found in *L. albus* cv. Estoril. No differences were observed between the other two species.

**Table 3:** Effect of species, sowing date, and location on the content of essential macro (g kg^−1^ dry matter, DM) and trace (mg kg^−1^ DM) elements in the studied *Lupinus* species.

	Ca	P	K	Mg	Na	Mn	Fe	Co	Cu	Zn	Mo
Species											
*Lupinus albus* cv. Estoril	6.06^a^	1.99^a^	11.6^a^	1.85^a^	1.09^a^	2,195^b^	134.2^a^	1.62^b^	9.33	29.1^a^	0.492
*Lupinus angustifolius* cv. Tango	10.4^b^	2.55^b^	16.0^b^	2.92^c^	1.33^b^	401.2^a^	220.0^c^	2.02^c^	9.33	59.9^b^	0.528
*Lupinus luteus* cv. Cardiga	6.76^a^	2.53^b^	20.6^c^	2.44^b^	1.42^b^	365.0^a^	160.3^b^	1.15^a^	9.43	58.5^b^	0.552
Sowing date											
D1	8.02	2.33	16.3	2.48	1.37	1106	180.5	1.55	8.94^a^	54.0	0.602
D2	8.12	2.31	16.3	2.46	1.35	1068	181.5	1.68	9.26^a,b^	48.4	0.498
D3	7.45	2.37	15.2	2.31	1.19	824.8	150.8	1.70	9.31^a,b^	42.0	0.502
D4	7.40	2.42	16.4	2.36	1.20	949.2	173.3	1.46	9.94^b^	52.3	0.494
Location											
Mirandela	7.31	3.18	18.0	2.62	1.37	1107	144.6	0.587	7.74	45.8	0.715
Vila Real	8.18	1.54	14.1	2.18	1.19	867.3	198.4	2.61	11.0	52.6	0.332
Statistics											
*p*-Values											
Species	<0.001	<0.001	<0.001	<0.001	0.013	<0.001	<0.001	<0.001	0.889	<0.001	0.342
Sowing date	0.196	0.649	0.215	0.127	0.362	0.092	0.069	0.481	0.009	0.141	0.074
Location	0.004	<0.001	<0.001	<0.001	0.059	0.006	<0.001	<0.001	<0.001	0.085	<0.001
Species × sowing date	0.420	0.257	0.398	0.031	0.342	0.028	0.826	0.848	0.047	0.337	0.066
Species × location	<0.001	<0.001	<0.001	<0.001	0.894	<0.001	<0.001	<0.001	<0.001	0.003	0.007
Sowing date × location	0.359	0.204	0.602	0.417	0.335	0.022	<0.001	0.534	0.330	0.124	0.044
RSD	1.605	0.355	2.41	0.317	0.505	464.07	49.64	0.690	1.129	21.21	0.183
R^2^	0.705	0.889	0.812	0.789	0.218	0.835	0.656	0.772	0.815	0.476	0.639
Adjusted R^2^	0.641	0.864	0.771	0.743	0.047	0.799	0.581	0.722	0.775	0.362	0.560
MTL											
Cattle	15	7	20	6	45 (NaCl)	2000	500	25	40	500	5
Sheep	15	6	20	6	40 (NaCl)	2000	500	25	15	300	5

MTL is the maximum tolerable level in the feed based on indexes of animal health [40]. RSD, residual standard deviation; R^2^, coefficient of determination. ^a,b^Means within a column with different superscript letters are significantly different (*p* < 0.05).

The highest manganese (Mn) content was observed for *L. albus* cv. Estoril, particularly on the first, second, and fourth sowing dates (*p* = 0.028; Fig. S4E) and when sown in Mirandela (*p* < 0.001; Fig. S3I). Additionally, regardless of the species, Mn content was higher at the first sowing date in Mirandela (*p* = 0.022; Fig. S2F). The iron (Fe) content of *L. angustifolius* cv. Tango was higher when sown in Vila Real than in Mirandela, with no differences being observed between locations for the other two species studied (*p* < 0.001; Fig. S3J). Regardless of the species, Fe content was higher on the fourth sowing date in Vila Real and lower on the third and fourth sowing dates in Mirandela (*p* < 0.001; Fig. S2G). Co content was higher in Vila Real than in Mirandela, with differences between locations being greater for *L. angustifolius* cv. Tango (*p* < 0.001; Fig. S3K). Copper (Cu) content was higher on the fourth sowing date for *L. albus* cv. Estoril, with no differences between sowing dates for the other two species of *Lupinus* (*p* = 0.047; Fig. S4F). In addition, when sown in Vila Real, *L. albus* cv. Estoril and *L*. *angustifolius* cv. Tango had a higher Cu content than when sown in Mirandela (*p* < 0.001; Fig. S3L). Zinc (Zn) content did not differ between locations for *L. luteus* cv. Cardiga and *L*. *albus* cv. Estoril but was higher in *L. angustifolius* cv. Tango sown in Mirandela than in Vila Real (*p* = 0.003; Fig. S3M). Molybdenum (Mo) content was higher when *Lupinus* was sown in Mirandela than in Vila Real, in particular for *L. albus* cv. Estoril (*p* = 0.007; Fig. S3N). The sowing date did not affect Mo content when sown in Vila Real, but when sown in Mirandela, Mo content was higher on the first sowing date (*p* = 0.044; Fig. S2H). Selenium (Se) was only detected in some plots of *L. albus* cv. Estoril and *L*. *angustifolius* cv. Tango, namely, sown in Vila Real, with values that varied between 0.085 and 0.156 mg kg^−1^ DM.

The interaction between species and location (*p* < 0.05; Table 4; Fig. S3O–S3R; Table S5) affected the levels of the toxic elements rubidium (Rb), strontium (Sr), Cd, Pb, and Hg (only detected in some plots sown in Vila Real). Arsenic (As) was only detected in some plots of the Estoril and Tango cultivars sown in Vila Real on the third and fourth dates, with values between 0.413 and 0.516 mg kg^−1^ DM. The content of toxic elements (chromium, Li, titanium, nickel, cesium, Ba, thallium, beryllium, and antimony) is presented in Table S5.

**Table 4:** Effect of species, sowing date, and location on toxic elements (mg kg^−1^ dry matter) content in the studied *Lupinus* species.

	Rb	Sr	Cd	Pb	Hg
Species					
*Lupinus albus* cv. Estoril	57.7^a^	19.4^a^	0.027^a^	0.498^a^	0.040^b^
*Lupinus angustifolius* cv. Tango	56.3^a^	39.5^c^	0.073^b^	0.760^b^	0.032^a^
*Lupinus luteus* cv. Cardiga	78.1^b^	22.5^b^	0.131^c^	0.476^a^	0.029^a^
Sowing date					
D1	64.7	28.6	0.082	0.662	0.029^a^
D2	63.5	27.6	0.079	0.572	0.041^c^
D3	63.1	26.3	0.070	0.526	0.030^a,b^
D4	64.9	26.1	0.078	0.552	0.035^b,c^
Location					
Mirandela	39.0	29.5	0.070	0.183	–
Vila Real	89.1	24.8	0.084	0.973	–
Statistics					
*p*-Values					
Species	<0.001	<0.001	<0.001	<0.001	0.009
Sowing date	0.903	0.443	0.446	0.189	0.016
Location	<0.001	<0.001	0.011	<0.001	–
Species × sowing date	0.722	0.525	0.285	0.797	0.283
Species × location	<0.001	0.004	0.004	<0.001	–
Sowing date × location	0.650	0.499	0.883	0.775	–
RSD	11.47	6.62	0.0302	0.251	0.009
R^2^	0.878	0.722	0.733	0.797	0.502
Adjusted R^2^	0.851	0.661	0.675	0.753	0.283
MTL					
Cattle	–	2000	10	100	-inorganic/2 organic
Sheep	–	2000	10	100	-inorganic/2 organic

MTL is the maximum tolerable level in the feed based on indexes of animal health [40]. RSD, residual standard deviation; R^2^, coefficient of determination. ^a,b,c^Means within a column with different superscript letters are significantly different (*p* < 0.05).

### 3.3 Alkaloid profile

**Fig. 3:**
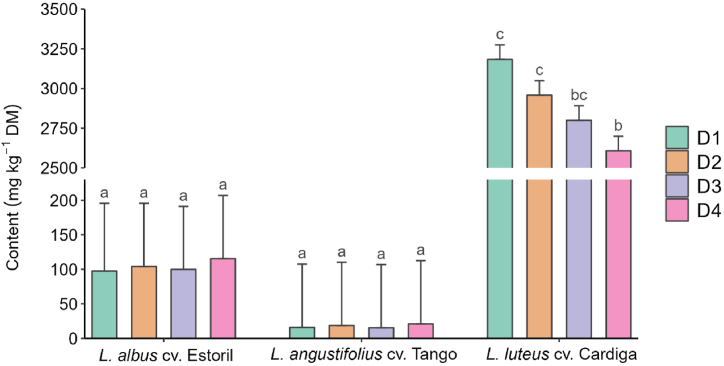
Effect of interaction between species and sowing date on total alkaloid content in different *Lupinus* species. ^a,b,c,d^Means with different superscript letters are significantly different (*p* < 0.05).

The effects of lupin species, sowing dates, and locations and their interactions on the alkaloid profile are presented in Table 5, Table 6, Table 7, with the means and standard deviation presented as supplementary material (Tables S6–S8). Total alkaloid content was not affected by location (*p* > 0.05; Table 5), but for *L. luteus* cv. Cardiga, it decreased from the first to the fourth sowing date, with values higher than those observed for the other studied species, which were not affected by sowing date (*p* = 0.036; Fig. 3). Total indole alkaloids were detected only in *L. luteus* cv. Cardiga (Table 5), with the highest values observed in Mirandela from the second to the fourth sowing dates; no differences were observed between sowing dates in Vila Real (Fig. S2I). Among sowing dates, total piperidine alkaloids were the highest on the first date for *L. luteus* cv. Cardiga, with no differences between dates for *L. albus* cv. Estoril and *L*. *angustifolius* cv. Tango (Fig. S4G), and higher on the first sowing date in Mirandela compared with other dates in both locations that did not differ between them (Fig. S2J). Total bicyclic alkaloids were detected only in *L. luteus* cv. Cardiga, especially when sown in Vila Real (Table 5). The content of tetracyclic alkaloids was higher when *L. luteus* cv. Cardiga was sown in Vila Real than in Mirandela, with the opposite being observed for the other two species studied (Fig. S3S) and for the third sowing date, with no differences observed between sowing dates for the other two species (Fig. S4H).

**Table 5:** Effect of species, sowing date, and location on the concentrations (mg kg^−1^ dry matter) of indole, piperidine, quinolizidine (bicyclic and tetracyclic), and total alkaloids in the studied *Lupinus* species.

	Quinolizidine				
	Indole	Piperidine	Bicyclic	Tetracyclic	Total
Species					
*Lupinus albus* cv. Estoril	–	32.6^a^	–	70.7^b^	104^a^
*Lupinus angustifolius* cv. Tango	–	2.94^a^	–	14.8^a^	17.7^a^
*Lupinus luteus* cv. Cardiga	354	238^b^	2039	256^c^	2,887^b^
Sowing date					
D1	305^a^	134^b^	2263	107	1099
D2	343^ab^	72.3^a^	2189	111	1027
D3	385^b^	75.1^a^	1945	120	972
D4	384^b^	83.2^a^	1760	117	915
Location					
Mirandela	430	86.2	1895	115	976
Vila Real	279	96.2	2183	113	1030
Statistics					
*p*-Values					
Species	–	<0.001	–	<0.001	<0.001
Sowing date	0.003	0.014	0.053	0.211	0.010
Location	<0.001	0.503	0.039	0.678	0.309
Species × sowing date	–	0.001	–	0.047	0.036
Species × location	–	0.268	–	<0.001	0.064
Sowing date × location	0.050	0.005	0.089	0.052	0.120
RSD	51.6	80.3	441	24.6	288
R^2^	0.787	0.728	0.390	0.958	0.964
Adjusted R^2^	0.725	0.668	0.213	0.949	0.956

RSD, residual standard deviation; R^2^, coefficient of determination. ^a,b^Means within a column with different superscript letters are significantly different (*p* < 0.05).

**Table 6:** Effect of species, sowing date, and location on the concentrations (mg kg^−1^ dry matter) of individual indole, piperidine, and tricyclic quinolizidine alkaloids in different *Lupinus* species.

	Indole		Piperidine		Bicyclic		
	Gramine	Gramine derivative	Smipine	Ammodendrine	Lupinine	Lusitanine	Feruloyllupinine
Species							
*Lupinus albus* cv. Estoril	–	–	24.1	8.48^a^	–	–	–
*Lupinus angustifolius* cv. Tango	–	–	–	2.94^a^	–	–	–
*Lupinus luteus* cv. Cardiga	330	25.5	–	238^b^	2034	52.4	12.3
Sowing date							
D1	292^a^	18.3^a^	22.2^a^	127^b^	2179	74.7^c^	13.7
D2	314^a^	28.5^b^	20.7^a^	65.4^a^	2124	59.6^b^	8.35
D3	362^b^	23.1^ab^	23.9^a^	67.1^a^	1898	34.0^a^	15.9
D4	352^b^	32.2^b^	29.5^b^	73.3^a^	1935	41.1^a^	11.4
Local							
Mirandela	400	31.5	29.6	76.3	1945	58.2	8.74
Vila Real	260	19.6	18.6	90.0	2132	46.6	15.9
Statistics							
*p*-Values							
Species	–	–	–	<0.001	–	–	–
Sowing date	0.006	0.039	0.022	0.013	0.116	<0.001	0.772
Location	<0.001	0.001	<0.001	0.359	0.067	0.006	0.169
Species × sowing date	–	–	–	0.001	–	–	–
Species × location	–	–	–	0.382	–	–	–
Sowing date × location	0.060	0.300	0.218	0.004	0.114	0.002	0.681
RSD	47.8	10.5	6.51	80.1	304	13.0	13.8
R^2^	0.782	0.484	0.592	0.743	0.342	0.741	0.243
Adjusted R^2^	0.718	0.320	0.468	0.686	0.142	0.666	−0.199

RSD, residual standard deviation; R^2^, coefficient of determination. ^a,b^Means within a column with different superscript letters are significantly different (*p* < 0.05).

**Table 7:** Effect of species, sowing date, and location on the contents (mg kg^−1^ dry matter) of individual tetracyclic quinolizidine alkaloids in different *Lupinus* species.

	Tetracyclic								
	α-*Iso*-sparteine	Sparteine	β-*Iso*-sparteine	Dehydrosparteine	Hydroxy-β-isosparteine	Multiflorine	Lupanine	7-Hydroxylupanine	13-α-Angelolyoxylupanine
Species									
*Lupinus albus* cv. Estoril	–	20.0^a^	–	–	–	3.70	30.8^b^	13.6	2.89
*Lupinus angustifolius* cv. Tango	–	4.27^a^	–	–	–	–	12.7^a^	–	–
*Lupinus luteus* cv. Cardiga	7.24	232^b^	3.85	4.16	7.90	–	<2.57	–	–
Sowing date									
D1	7.90	85.0	2.98	4.81	9.35	3.88	14.6	8.13^a^	2.90^ab^
D2	6.77	80.3	5.24	3.82	5.78	3.96	16.8	12.5^a^	2.13^a^
D3	7.57	87.6	3.75	4.37	8.92	<2.57	13.6	21.0^b^	3.19^b^
D4	6.72	89.0	3.43	3.65	7.55	4.66	14.0	12.7^a^	3.36^b^
Location									
Mirandela	6.65	76.0	3.85	3.43	6.93	4.43	21.1	23.3	3.68
Vila Real	7.83	94.9	3.86	4.90	8.87	2.98	8.40	3.91	2.10
Statistics									
*p*-Values									
Species	–	<0.001	–	–	–	–	<0.001	–	–
Sowing date	0.581	0.792	0.370	0.293	0.108	0.055	0.293	<0.001	0.019
Location	0.109	0.004	0.990	0.003	0.078	0.021	<0.001	<0.001	<0.001
Species × sowing date	–	0.038	–	–	–	–	0.350	–	–
Species × location	–	<0.001	–	–	–	–	<0.001	–	–
Sowing date × location	0.048	0.045	0.037	0.001	0.172	0.241	0.135	<0.001	0.242
RSD	2.32	25.2	3.05	1.45	3.51	1.93	6.91	5.01	0.855
R^2^	0.321	0.961	0.289	0.557	0.325	0.377	0.851	0.874	0.645
Adjusted R^2^	0.114	0.949	0.082	0.416	0.128	0.188	0.818	0.837	0.532

RSD, residual standard deviation; R^2^, coefficient of determination. ^a,b^Means within a column with different superscript letters are significantly different (*p* < 0.05).

**Fig. 4:**
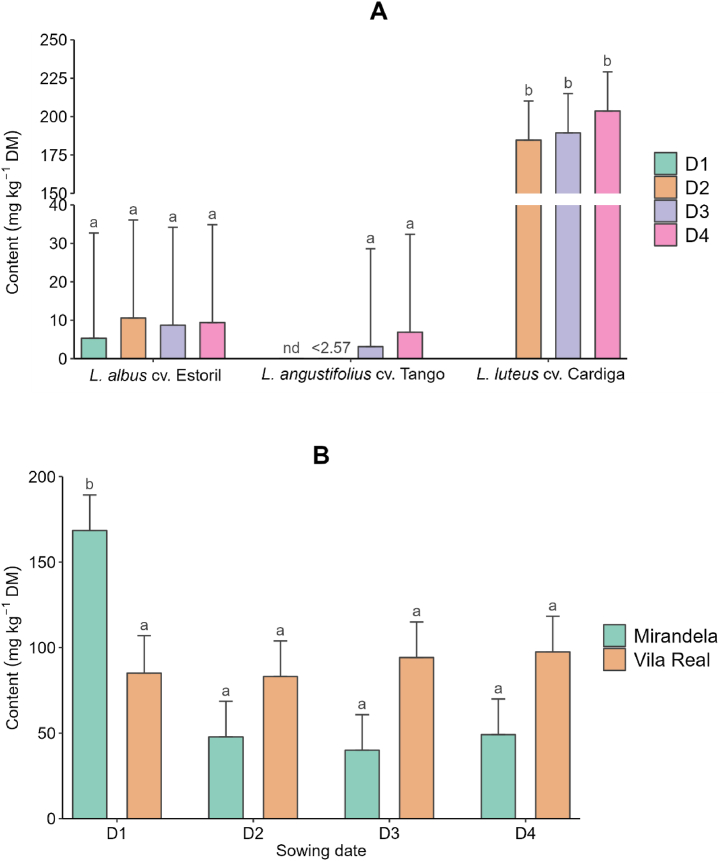
Effect of interactions between species and sowing date (A) and sowing date and location (B) on ammodendrine content in different *Lupinus* species. ^a,b^Means within each panel with different superscript letters are significantly different (*p* < 0.05). nd means not detected. <(value) means lower than the limit of quantification.

**Fig. 5:**
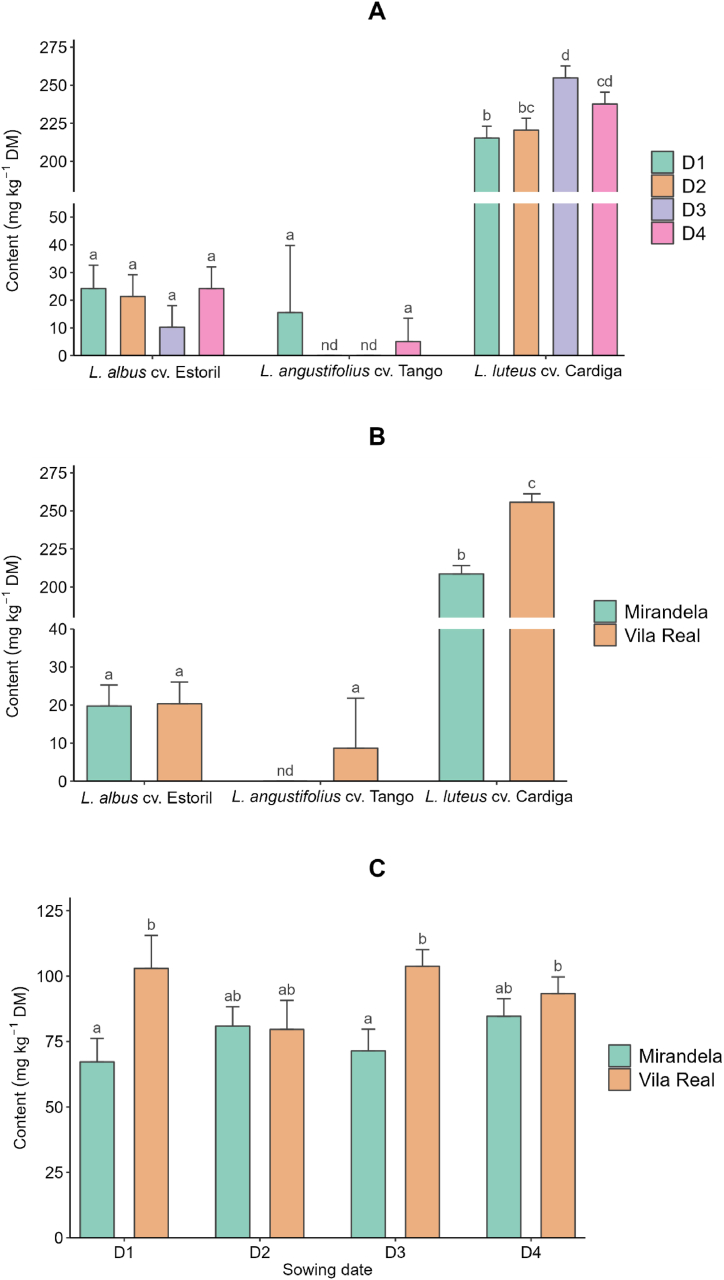
Effect of interactions between species and sowing date (A), species and location (B), and sowing date and location (C) on sparteine content in different *Lupinus* species. ^a,b^Means within each panel with different superscript letters are significantly different (*p* < 0.05). nd means not detected.

**Fig. 6:**
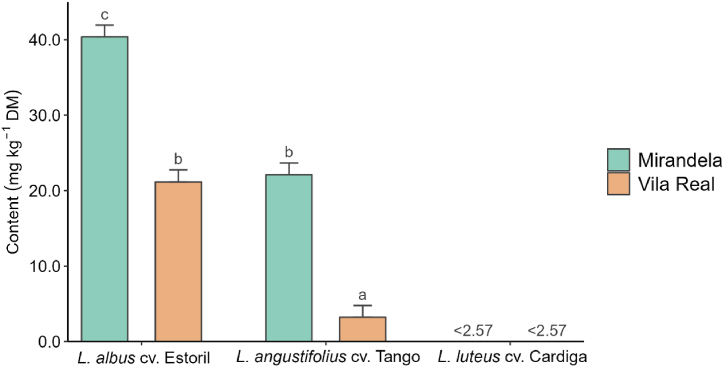
Effect of interaction between species and location on lupanine content in different *Lupinus* species. ^a,b,c^Means with different superscript letters are significantly different (*p* < 0.05). <(value) means lower than the limit of quantification.

Table 6 presents the effects of species, sowing date, and location on individual indole, piperidine, and bicycle and tricyclic quinolizidine alkaloid concentrations. Gramine and gramine derivatives, only detected in *L. luteus* cv. Cardiga, were lower on the first sowing date and in Vila Real. Smipine, only found in *L. albus* cv. Estoril, was the highest at the fourth sowing date and in Mirandela. The sowing date did not affect the ammodendrine content of *L. albus* cv. Estoril and *L*. *angustifolius* cv. Tango, but it was the highest at the first sowing date for *L. luteus* cv. Cardiga (Fig. 4A) and in Mirandela on the first sowing date (Fig. 4B). Lupinine, lusitanine, and feruloyllupinine, detected only in *L. luteus* cv. Cardiga, were not affected by sowing date or location, except for the lusitanine content, which was higher on the first sowing date both in locations and on the second date in Mirandela (Fig. S2K). Regarding the individual tetracyclic quinolizidine alkaloids (Table 7), only sparteine and lupanine were detected in all *Lupinus* species studied, the former being higher in *L. luteus* cv. Cardiga and the latter in *L. albus* cv. Estoril. The sparteine content of *L. luteus* cv. Cardiga was affected by the sowing date and location, but it was similar between dates and locations for the other two species (Fig. 5A and B). Regardless of the species, the sparteine content was higher in Mirandela on the first and third dates (Fig. 5C). Lupanine content was higher in Mirandela than in Vila Real for *L*. *albus* cv. Estoril and *L*. *angustifolius* cv. Tango (Fig. 6). α-*Iso*-sparteine, β-*iso*-sparteine, dehydrosparteine, and hydroxy-β-isosparteine were detected only in *L. luteus* cv. Cardiga, being differently affected by the interaction between sowing date and location (Fig. S2L–S2N). Multiflorine, 7-hydroxylupanine, and 13-α-angelolyoxylupanine were only detected in *L. albus* cv. Estoril, with the highest values of multiflorine and 13-α-angelolyoxylupanine found in Mirandela (Table 7). 7-Hydroxylupanine content was highest when sown in Mirandela on the third date, intermediate for the other dates in the same location, and lower in Vila Real, regardless of the sowing date (Fig. S2O).

### 3.4 Effect of environmental conditions on productivity, chemical composition, and alkaloid content

**Fig. 7:**
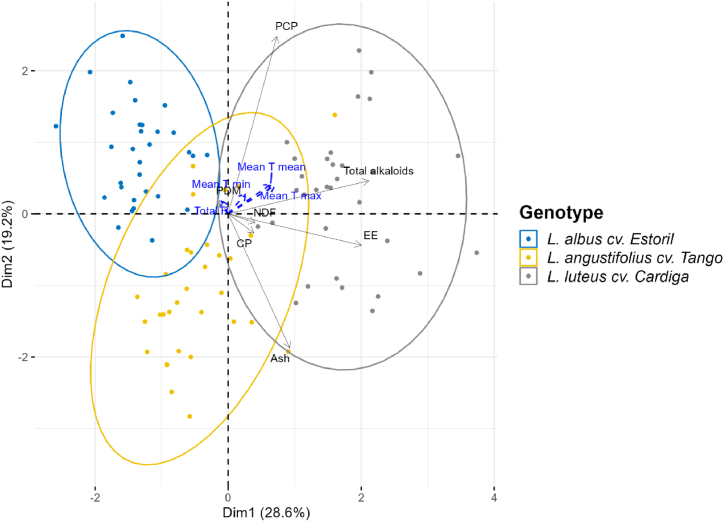
Principal component analysis (PCA) biplot of *Lupinus* forage showing the relationship between genotypes, environmental variables, productivity, proximate chemical composition, and total alkaloids. PDM, production of dry matter; PCP, production of crude protein; NDF, neutral detergent fiber; CP, crude protein; EE, ether extract; Mean T mean, mean of the monthly mean temperatures; Mean T min, mean of the minimum monthly temperature; Mean T max, mean of the maximum monthly temperature; Total R, total rainfall.

The correlation pattern between genotypes and environmental conditions was characterized by PCA analysis (Fig. 7), considering the productivity indexes, the proximate chemical composition, and the total alkaloid content. The first two components accounted for 47.8% of the total variability of the biplot, showing a distinction between genotypes mainly related to the production of CP (PCP), ash, EE, and total alkaloid contents. From the studied environmental factors, mean temperatures were those with a higher positive correlation with PCP and total alkaloid content.

**Fig. 8:**
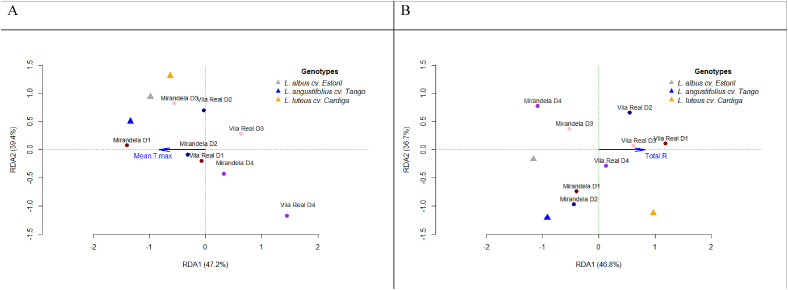
Relationship between genotypes, environments (location × sowing date), and environmental factors by RDA analysis of (A) production of dry matter and (B) total alkaloid content. Mean T max, mean of the maximum monthly temperature; Total R, total rainfall.

To understand which environmental factors are responsible for the differences in productivity and total alkaloid content between genotypes, an RDA analysis was conducted (Fig. 8). For the production of DM (PDM), the environmental variable with the most significant impact was the average maximum temperature (*p* = 0.022, model adjusted R^2^ = 0.384, Fig. 8A). By contrast, for the total alkaloid content, the total rainfall was found to be significant (*p* = 0.007, model adjusted R^2^ = 0.379, Fig. 8B). For the PCP, no environmental variable was considered to be significant. The mean maximum temperatures associated essentially with Mirandela and the earliest sowing dates had an impact on higher PDM for all the genotypes. The higher total rainfall observed at Vila Real when compared with Mirandela was responsible for the higher alkaloid content.

## 4 Discussion

Among the numerous species of the genus from the Old World Group, only *L. angustifolius*, *L. albus*, and *L. luteus* are cultivated [41]. Here, three species of lupins (*L. albus* cv. Estoril, *L. angustifolius* cv. Tango, and *L. luteus* cv. Cardiga) were sown in two different locations (Mirandela and Vila Real) on four different sowing dates, from early to late autumn, and harvesting occurred in May. Lupin species, sowing date, and location, as well as their interactions, significantly affected the vast majority of measured parameters, emphasizing the effects of climate and soil conditions on these crops, as previously reported [42].

### 4.1 Forage production, nutritive value, and mineral profile

In accordance with previous studies [[43], [44], [45]], the forage production obtained was 2.74, 4.48, and 4.86 t DM ha^−1^ for *L. angustifolius* cv. Tango, *L. luteus* cv. Cardiga, and *L. albus* cv. Estoril, respectively, having been affected by sowing date and location, with higher production being observed in Mirandela and on earlier sowing dates, reflecting different climatic and soil conditions. In fact, the mean maximum temperature showed a positive impact on forage productivity, higher at Mirandela at early sowing dates.

Although lupins are able to grow in acidic soils [46], the forage production differences between the two locations can be explained by the soil characteristics. In fact, compared with Mirandela, the Vila Real soil is more acidic (pH 4.8), with lower levels of P and K and extractable bases, and contains Al. Acidic soils usually make P inaccessible to plants [47] and release phytotoxic Al cations, greatly hampering crop production. In several lupin species, P deficiency and Al toxicity induce the formation of cluster roots [48], which cause rhizosphere acidification by a high cation/anion uptake ratio and the excretion of organic acids and provide an enlarged surface for interaction with the rhizosphere soil, promoting the mobilization of P and other nutrients, the symbiotic relationship with soil bacteria (*e.g.*, *Bradyrhizobium* genus), and contributing to Al immobilization [49,50].

Although there is a well-known effect of species and maturation stage on mineral profiles, the content of macro and trace elements observed in the present study was comparable to that found for three wild species of lupins (*L. exaltatus*, *L. mexicanus*, and *L. rotundiflorus*) cultivated in Mexico under irrigation and harvested at different stages of maturity [51], all below the maximum tolerable levels of feeds for cattle and sheep [40]. In addition, the concentration of individual minerals in forages depends on their content in the soil, as observed in the present study for P and K, which were higher in lupins grown in Mirandela, reflecting the higher content of these elements in the soil of this location. However, the Ca content in *L. angustifolius* cv. Tango and *L*. *albus* cv. Estoril could not be explained by the nutrient availability in the soil at the two locations. In this case, as shown by studies in white lupins [50,52], Ca concentration must have been affected by P nutritional status of plants. Low P supply increases cluster root formation in lupin plants, and these roots play an important role in the acquisition of not only P but also other mineral elements, resulting, *e.g*., in increased Ca and Mg concentrations and reduced S concentrations in roots and shoot tissues [50,52].

When regarding forage as a primary mineral source for ruminant animals, attention should be paid to other essential elements such as Se, whose concentration in the studied lupin species was extremely low. This constitutes a challenge in many regions of the world where Se concentrations in soils and locally produced forages are low [53]. As in both locations of the present study, dietary supplementation is necessary to prevent deficiency symptoms. Moreover, the release of carboxylates by lupins makes these legumes relevant in phytoremediation, promoting the decontamination of soil environments [54], namely, by taking up heavy metals such as Zn, Cd, As, and Pb [55,56]. Here, the soil in both locations was not contaminated, thus the levels of toxic elements in the three studied species were low and not considered harmful for cattle and sheep [40].

Despite the differences observed between treatments, all lupins studied showed a high moisture content and low nonstructural carbohydrate content at harvest, which, together with the well-known relatively high buffering capacity of legumes [57], do not allow their direct ensiling, requiring wilting and/or the use of an ensiling additive to prevent fermentation losses and minimize the production of effluents [44,58]. However, the thick and fibrous stems of lupins make wilting difficult [58], so the use of conditioning devices may be necessary to accelerate wilting and decrease effluent production and respiration losses. This approach may also be advantageous to decrease leaf losses when conservation as hay is considered. Lupins/cereals bi-crops or intercropping are other approaches that have been shown to increase DM yield, with triticale having the advantage of being less competitive than other cereals. In fact, Dawson [16] reported an increase in DM content from 161 g kg^−1^ when lupins were grown separately to 351 g kg^−1^ when lupins and triticale were sown as a mixture.

The relatively high digestibility associated with the higher protein content of the studied lupin species than ryegrass and winter-cereal silages [59,60] makes them interesting forages to be used as sustainable protein sources in diets for ruminant animals, including for high-producing animals, namely, in diets based on maize silage for dairy cows. This assumes particular importance in the current context of Europe's high dependence on protein sources, making it highly vulnerable to the international market, and is in line with the 10.13039/100016124Common Agricultural Policy measures that have sought to support European protein crops to address the imbalance between supply and demand for plant proteins [1].

### 4.2 Alkaloid profile

The European Food Safety Authority (EFSA) released a Scientific Opinion on the risk of quinolizidine alkaloids in food and feed, and based on a margin of safety approach, the risk of acute poisoning was considered low when <0.16 mg of sparteine per kg of body weight of quinolizidine alkaloids was ingested [25]. Moreover, EFSA stated the existence of indirect evidence of a possible transfer of quinolizidine alkaloids from feed to milk that would thus constitute an additional exposure source. In fact, Engel et al. [61] recently demonstrated that with the administration of only 1 kg DM day^−1^ of sweet *L. angustifolius* (containing 1774 mg of total quinolizidine alkaloids kg^−1^ DM), there was a transfer of quinolizidine alkaloids into the milk, resulting in a total concentration of 2.81 mg kg^−1^ milk, with transfer rates ranging from 0.13% for sparteine to 3.74% for multiflorine, thus suggesting a potential health concern for high milk consumers. Additionally, alkaloids can be transferred to the nearby environment and exported to drainage water, reaching new crops and even animals drinking that contaminated water [20].

The alkaloid profile is known to be species-specific. In *L*. *albus* and *L. angustifolius*, lupanine is generally the most abundant quinolizidine alkaloid. Conversely, in *L. luteus*, the most common quinolizidine alkaloid is lupinine, together with sparteine, which has a low lupanine content [36,62]. Here, lupanine, smipine, and sparteine were the most abundant quinolizidine alkaloids in *L. albus* cv. Estoril, lupanine, and sparteine in *L. angustifolius* cv. Tango, and lupinine, gramine, ammodendrine, and sparteine in *L. luteus* cv. Cardiga. There are only a few studies on the toxicity of quinolizidine alkaloids in ruminant animals, with severe toxic effects only being described for the teratogenic quinolizidine alkaloid anagyrine [63]. Information on other quinolizidine alkaloids is scarce and mainly focuses on zootechnical performance. It has been reported that there is decreased feed intake [64,65] and no degradation of sparteine in bovine and ovine ruminal fluid [25]. Considering that an intake of 4.5 mg of total alkaloids per kg^−1^ body weight is tolerated by heifers [25] and an intake of 5.45 mg of quinolizidine alkaloids per kg^−1^ body weight is tolerated by dairy cows [61], the dietary inclusion of forages of *L. albus* cv. Estoril and *L*. *angustifolius* cv. Tango does not pose a risk to the animals, but the alkaloid levels in *L. luteus* cv. Cardiga compromise its inclusion level in the diet. In fact, the maximum inclusion without adverse health effects would be set at 1.6 and 1.5 kg DM of *L. luteus* cv. Cardiga forage, respectively, for a 400 kg heifer and a 650 kg dairy cow. Along with the dietary inclusion level, the utilization of lupins as protein crops in animal feeding implies the choice of adequate cultivars and agricultural operations and the application of postproduction processes following good agricultural practices [66]. In fact, traditionally, the seeds from the Cardiga cultivar are only used in animal feed after maceration with running water to decrease alkaloid content. Conversely, pod shells and straws do not pose any risk for the animals due to their low-alkaloid content [37]. Despite the constraints of this cultivar, its high adaptation and resilience to temperate regions and less fertile soils make it of high interest to be used as green manure, namely, to improve soil fertility for the subsequent crop, thus continuing to be an appreciated cultivar for the farmers.

Along with the genotype, the level and pattern of alkaloids depend on edaphoclimatic conditions, as observed in the present study by the significant interactions between lupin species, sowing date, and location. Among climatic factors, temperature, light, and drought stress are considered the most relevant factors. The synthesis of quinolizidine alkaloids follows a diurnal light-regulated cycle with maximum levels at noon or early afternoon and minimum levels during the night [18], increasing with increasing temperatures [67] and with early drought stress [68]. Soil characteristics also affect the quinolizidine alkaloid content, which lowers with higher soil pH [69]. The effects of the presence or deficiency of several mineral elements, such as K, P, and N, on the quinolizidine alkaloid content and pattern depend on the species and varieties [18].

Overall, the results indicate that forage production is heightened by higher soil pH and temperature. Conversely, environmental stress tends to promote the alkaloid synthesis and accumulation that vary among the lupin species examined. In fact, forage production was heightened by both the higher maximum temperature (from March to May; Fig. 8A) and soil pH observed at Mirandela (20.3 °C and 18.6 °C and 6.1 and 4.8, respectively, for Mirandela and Vila Real). By contrast, alkaloid content is associated with rainfall that was the highest in Vila Real (41.7 and 64.8 mm, respectively, for Mirandela and Vila Real; Fig. 8B), as previously observed [42]. However, notably, the effect of edaphoclimatic conditions on alkaloid content also depends on the *Lupinus* species, as a significant interaction between *Lupins* species and location was observed for total tetracyclic quinolizidine alkaloids and a tendency for total alkaloids, with *L. luteus* cv. Cardiga presenting the highest alkaloid content when sown at Vila Real and *L*. *albus* cv. Estoril and *L*. *angustifolius* cv. Tango when sown at Mirandela. This emphasizes the different behaviors of the studied species depending on soil and climate conditions.

Despite being focused on lupin seeds, a recent literature review [70] studied the influence of biotic, abiotic, and genotypic factors on the production of quinolizidine alkaloids by lupins and suggested that, to avoid high alkaloid content in seeds, the optimal options are use of sweet cultivars with high concentrations of 13-hydroxylupanine and 13-tigloyloxylupanine to increase protection against pathogens; use of N-deficient fertilizers with 240 mg K kg^−1^ and 60 mg P kg^−1^ along with a high soil pH; provide a cold environment with high light exposure and standard daytime cycles and well-watering procedures; and consider an organic growing system. To the best of the authors’ knowledge, a systematic review on the modulation of alkaloid content in lupin forage has not been performed, emphasizing the need for further studies that allow for a more efficient use of lupins as a protein source that is well adapted to temperate regions and soils with lower fertility, with a relevant impact on livestock sustainability.

## 5 Conclusion

The present study highlighted the challenges and opportunities of three lupin species (*L. albus*, *L. angustifolius*, and *L. luteus*) for the livestock sector. It was shown that lupins have potential for forage production with high levels of protein in cultural systems with low inputs, low costs, and reduced environmental impact, proving to be an interesting option for the development of sustainable agricultural systems. The species examined exhibited different nutrient and alkaloid profiles under cultivation in the same agroclimatic conditions. Data generated might contribute to drive lupin users to follow an integrated strategy toward the improvement of temperate agricultural ecosystems, thus contributing to the rural economy and reducing desertification, a relevant issue in several regions of the world.

## Funding

This study received financial support from AgriFood XXI—Development and consolidation of research in the agrifood sector in Northern Portugal I&D&I project (NORTE-01-0145-FEDER-000041), cofinanced by the European Regional Development Fund through the NORTE 2020 program (Programa Operacional Regional do Norte 2014/2020), and from the Portuguese Foundation for Science and Technology (10.13039/501100001871FCT/10.13039/501100006111MCTES) through projects UIDB/04033/2020 (https://doi.org/10.54499/UIDB/04033/2020), UIDB/50006/2020 (https://doi.org/10.54499/UIDB/50006/2020), and UIDP/50006/2020 (https://doi.org/10.54499/UIDP/50006/2020). I.M. Valente and M.R.G. Maia acknowledge 10.13039/501100001871FCT for funding through program DL 57/2016—Norma transitória DL 57/2016/CP1346/CT0035 10.13039/100000201DOI 10.54499/DL57/2016/CP1346/CT0035 and SFRH/BPD/70176/2010, respectively).

## Data availability statement

Data will be made available on request.

## CRediT authorship contribution statement

**Ana R.J. Cabrita:** Writing – original draft, Methodology, Investigation, Formal analysis, Conceptualization. **Inês M. Valente:** Investigation, Formal analysis, Methodology, Writing – review & editing. **André Monteiro:** Methodology, Investigation. **Carla Sousa:** Formal analysis. **Carla Miranda:** Methodology, Investigation. **Agostinho Almeida:** Formal analysis. **Paulo P. Cortez:** Writing – review & editing. **Carlos Castro:** Investigation. **Margarida R.G. Maia:** Writing – review & editing, Formal analysis. **Henrique Trindade:** Writing – review & editing, Supervision, Investigation, Funding acquisition, Conceptualization. **António J.M. Fonseca:** Writing – review & editing, Supervision, Project administration, Methodology, Investigation, Funding acquisition, Conceptualization.

## Declaration of competing interest

The authors declare that they have no known competing financial interests or personal relationships that could have appeared to influence the work reported in this paper.
