# Supplementary material for: Environmental conditions affect the nutritive value and alkaloid profiles of Lupinus forage: Opportunities and threats for sustainable ruminant systems
Source: Heliyon. 2024 Mar 27;10(7):e28790. doi: 10.1016/j.heliyon.2024.e28790 (PMC11002601; doi:10.1016/j.heliyon.2024.e28790)
Supplement: Multimedia component 1 [file mmc1.docx]

**Environmental conditions affect the nutritive value and alkaloid profiles of *Lupinus* forage: Opportunities and threats for sustainable ruminant systems**

Ana R.J. Cabrita^a*^, Inês M. Valente^a,b^, André Monteiro^c^, Carla Sousa^a^, Carla Miranda^c^†, Agostinho Almeida^d^, Paulo P. Cortez^e^, Carlos Castro^c^, Margarida R.G. Maia^a^, Henrique Trindade^c^, António J.M. Fonseca^a^

*** Correspondence:** [arcabrita@icbas.up.pt](mailto:arcabrita@icbas.up.pt)

**Table S1**

Physicochemical characteristics of the soils^1^.

|  | Mirandela | Vila Real |
| --- | --- | --- |
| Chemical properties |  |  |
| pH (water) | 6.1±0.10 | 4.8±0.10 |
| pH (KCl) | 5.2±0.30 | 3.9±0.10 |
| Organic matter (g kg^-1^) | 11±2.1 | 14±0.7 |
| Extractable P (mg P_2_O_5_ kg^-1^) | 225±28.3 | 67±6.7 |
| Extractable K (mg K_2_O kg^-1^) | 115±16.7 | 86±8.3 |
| Exchangeable bases (cmolc kg^-1^) |  |  |
| …Ca | 4.8±0.42 | 2.5±0.34 |
| …Mg | 1.1±0.08 | 0.7±0.17 |
| …K | 0.5±0.51 | 0.3±0.01 |
| …Na | 0.13±0.030 | 0.12±0.010 |
| …Al | n.d. | 0.7±0.09 |
| Effective cation exchange capacity (ECEC) | 6.3±0.54 | 4.2±0.29 |
| Particle-size distribution (g kg^-1^) |  |  |
| Coarse sand (200 - 2000 μm) | 90±13.0 | 210±9.0 |
| Fine sand (20 - 200 μm) | 624±10.0 | 387±8.0 |
| Silt (2 - 20 μm) | 160±7.0 | 275±10.0 |
| Clay (<2 μm) | 124±13.0 | 126±6.0 |

^1^Values are presented as mean ± standard deviation (n = 4); n.d., not detected.

**Table S2**

Chemical composition^1^ (g 100 g^-1^ dry matter, DM), DM (DMD; g 100 g^-1^) and organic matter (OMD; g 100 g^-1^ DM) digestibility and estimated metabolizable energy (ME; MJ kg^-1^ DM) in the studied *Lupinus* species for the four sowing dates (D) of the two locations (MI, Mirandela; VR, Vila Real)^2^.

|  | DM (%) | Ash | CP | EE | NDF | ADF | ADL | NSC | DMD | OMD | ME |
| --- | --- | --- | --- | --- | --- | --- | --- | --- | --- | --- | --- |
| ***Lupinus albus* cv. Estoril** |  |  |  |  |  |  |  |  |  |  |  |
| MID1 | 19±8.5 | 4.8±0.18 | 20±7.4 | 0.76±0.073 | 46±2.9 | 36±0.5 | 6.8±0.72 | 29±7.1 | 73±2.8 | 72±2.8 | 12±1.0 |
| MID2 | 18±2.0 | 4.7±0.37 | 17±1.2 | 0.87±0.243 | 44±2.3 | 35±2.3 | 6.1±0.89 | 34±3.2 | 72±1.5 | 72±1.4 | 12±0.2 |
| MID3 | 22±2.3 | 4.6±0.36 | 17±0.4 | 0.54±0.135 | 42±2.6 | 33±1.9 | 5.6±0.63 | 35±2.7 | 75±1.2 | 74±1.0 | 12±0.2 |
| MID4 | 17±6.6 | 4.8±0.23 | 21±7.3 | 0.86±0.081 | 39±3.7 | 31±2.8 | 5.0±0.51 | 35±4.5 | 76±4.2 | 76±4.2 | 13±1.1 |
| VRD1 | 18±1.6 | 4.8±0.59 | 13±1.0 | 1.3±0.50 | 49±3.7 | 38±3.6 | 6.8±0.68 | 33±3.5 | 67±3.5 | 67±3.7 | 11±0.5 |
| VRD2 | 17±1.0 | 4.6±0.30 | 14±1.1 | 1.1±0.17 | 50±4.8 | 39±4.1 | 7.3±0.81 | 31±3.9 | 65±5.4 | 65±5.6 | 11±0.9 |
| VRD3 | 19±1.8 | 4.9±1.09 | 11±1.2 | 0.88±0.255 | 42±2.2 | 33±1.7 | 6.1±1.05 | 41±4.0 | 72±2.3 | 72±2.5 | 11±0.3 |
| VRD4 | 18±0.4 | 4.6±0.39 | 13±1.3 | 1.1±0.10 | 42±1.3 | 33±1.0 | 6.4±0.73 | 40±1.0 | 72±1.7 | 73±2.7 | 12±0.4 |
| ***Lupinus angustifolius* cv. Tango** |  |  |  |  |  |  |  |  |  |  |  |
| MID1 | 14±1.4 | 7.2±0.46 | 20±0.4 | 1.5±0.37 | 44±3.7 | 35±3.1 | 6.5±0.73 | 28±3.3 | 67±5.9 | 66±5.9 | 11±0.9 |
| MID2 | 15±2.7 | 7.2±1.09 | 17±1.5 | 1.4±0.28 | 46±2.9 | 36±1.8 | 6.3±0.24 | 29±1.5 | 70±4.0 | 69±4.0 | 12±0.6 |
| MID3 | 29±7.1 | 6.5±0.36 | 19±2.6 | 1.0±0.24 | 48±3.7 | 37±2.5 | 5.8±0.58 | 26±3.1 | 72±2.7 | 71±3.0 | 12±0.6 |
| MID4 | 24±5.1 | 6.7±0.48 | 19±3.2 | 1.1±0.11 | 46±4.5 | 36±2.3 | 5.3±0.51 | 28±2.1 | 73±1.6 | 72±1.4 | 12±0.4 |
| VRD1 | 18±0.7 | 7.2±0.80 | 11±1.5 | 1.0±0.12 | 46±3.0 | 36±2.9 | 6.6±0.70 | 35±2.9 | 66±3.7 | 65±3.9 | 10±0.5 |
| VRD2 | 17±0.8 | 7.2±0.59 | 13±0.4 | 1.0±0.28 | 45±2.7 | 35±2.5 | 6.8±0.41 | 34±2.1 | 66±1.6 | 65±1.6 | 11±0.2 |
| VRD3 | 19±0.8 | 6.8±1.82 | 11±1.1 | 0.83±0.215 | 46±1.7 | 35±1.6 | 6.6±0.69 | 36±1.9 | 68±2.7 | 67±2.8 | 11±0.4 |
| VRD4 | 18±1.9 | 7.6±0.71 | 12±1.4 | 1.1±0.12 | 42±1.3 | 32±1.5 | 6.4±0.71 | 37±1.2 | 72±1.7 | 71±1.5 | 11±0.2 |
| ***Lupinus luteus* cv. Cardiga** |  |  |  |  |  |  |  |  |  |  |  |
| MID1 | 13±3.3 | 7.8±0.30 | 19±2.1 | 1.7±0.50 | 45±3.6 | 37±1.5 | 7.1±0.78 | 26±1.7 | 71±2.4 | 70±2.5 | 12±0.5 |
| MID2 | 13±1.8 | 7.4±0.60 | 19±1.3 | 1.5±0.20 | 43±2.9 | 33±1.9 | 5.9±0.33 | 29±2.8 | 73±2.7 | 72±3.0 | 12±0.5 |
| MID3 | 23±5.0 | 6.7±0.48 | 18±0.9 | 1.9±0.73 | 45±2.8 | 35±1.6 | 6.0±0.85 | 29±3.1 | 73±1.2 | 72±1.5 | 12±0.3 |
| MID4 | 18±6.5 | 7.0±0.36 | 18±1.8 | 1.4±0.34 | 44±1.4 | 34±1.1 | 5.7±0.70 | 30±1.0 | 74±2.0 | 72±2.1 | 12±0.4 |
| VRD1 | 14±0.4 | 5.6±0.71 | 15±0.9 | 1.2±0.58 | 49±2.5 | 38±2.1 | 6.4±0.83 | 29±1.4 | 66±5.1 | 63±8.1 | 11±1.2 |
| VRD2 | 14±0.8 | 6.0±0.28 | 15±0.7 | 1.7±0.23 | 50±4.8 | 39±2.2 | 6.8±0.76 | 28±5.0 | 65±3.9 | 65±3.8 | 11±0.5 |
| VRD3 | 15±0.7 | 5.2±0.52 | 15±0.9 | 1.5±0.27 | 45±1.5 | 37±1.2 | 6.6±0.52 | 33±0.9 | 69±0.6 | 69±0.7 | 11±0.1 |
| VRD4 | 16±1.2 | 5.7±0.52 | 15±1.1 | 1.9±0.23 | 47±3.9 | 35±0.9 | 6.1±0.79 | 30±3.7 | 70±0.5 | 70±0.4 | 11±0.1 |

^1^CP, crude protein; EE, ether extract; NDF, neutral detergent fibre; ADF, acid detergent fibre; ADL, acid detergent lignin; NSC, non-structural carbohydrates. NSC calculated as DM-ash-CP-EE-NDF. Metabolizable energy estimated as according to Givens *et al.* (1990): ME (MJ kg^-1^ DM) = 0.37 + 0.0142 OMD (g kg^-1^) + 0.0077 CP (g kg^-1^ DM).

^2^Values are presented as mean ± standard deviation (n = 4).

**Table S3**

Production (t ha^-1^) of forage dry matter (PDM), crude protein (PCP), digestible dry matter (PDMD), digestible organic matter (POMD), and metabolizable energy (PME, GJ ha^-1^) of the studied *Lupinus* species for the four sowing dates (D) of the two locations (MI, Mirandela; VR, Vila Real)^1^.

|  | PDM | PCP | PDMD | POMD | PME |
| --- | --- | --- | --- | --- | --- |
| ***Lupinus albus* cv. Estoril** |  |  |  |  |  |
| MID1 | 6.8±3.19 | 1.3±0.47 | 4.9±2.26 | 4.9±2.24 | 82±35.8 |
| MID2 | 6.7±3.16 | 1.1±0.50 | 4.8±2.38 | 4.8±2.36 | 79±38.5 |
| MID3 | 6.2±1.78 | 1.1±0.32 | 4.6±1.28 | 4.6±1.27 | 76±21.2 |
| MID4 | 3.4±1.29 | 0.67±0.261 | 2.6±0.94 | 2.5±0.92 | 42±15.0 |
| VRD1 | 5.6±2.31 | 0.69±0.276 | 3.8±1.66 | 3.8±1.65 | 61±26.4 |
| VRD2 | 5.5±1.94 | 0.77±0.232 | 3.6±1.11 | 3.5±1.10 | 58±18.1 |
| VRD3 | 2.8±1.49 | 0.31±0.156 | 2.0±1.04 | 2.0±1.03 | 32±16.3 |
| VRD4 | 1.8±0.60 | 0.24±0.079 | 1.3±0.42 | 1.3±0.46 | 22±7.3 |
| ***Lupinus angustifolius* cv. Tango** |  |  |  |  |  |
| MID1 | 2.2±1.08 | 0.44±0.203 | 1.4±0.62 | 1.4±0.61 | 24±10.6 |
| MID2 | 4.3±1.48 | 0.73±0.214 | 3.0±0.89 | 3.0±0.89 | 49±14.8 |
| MID3 | 4.3±2.43 | 0.79±0.399 | 3.1±1.62 | 3.0±1.59 | 50±26.4 |
| MID4 | 3.1±2.07 | 0.54±0.328 | 2.2±1.48 | 2.2±1.46 | 36±23.9 |
| VRD1 | 3.3±3.12 | 0.34±0.274 | 2.3±2.27 | 2.2±2.24 | 35±35.0 |
| VRD2 | 2.6±1.84 | 0.32±0.223 | 1.7±1.21 | 1.7±1.19 | 27±19.3 |
| VRD3 | 1.5±1.22 | 0.17±0.150 | 1.0±0.89 | 1.0±0.87 | 16±14.0 |
| VRD4 | 0.37±0.103 | 0.04±0.014 | 0.26±0.068 | 0.26±0.068 | 4.1±1.11 |
| ***Lupinus luteus* cv. Cardiga** |  |  |  |  |  |
| MID1 | 5.7±4.40 | 1.0±0.70 | 4.0±3.06 | 4.0±3.03 | 66±50.0 |
| MID2 | 3.8±3.20 | 0.71±0.572 | 2.7±2.29 | 2.7±2.25 | 45±37.5 |
| MID3 | 5.9±2.40 | 1.0±0.40 | 4.3±1.70 | 4.2±1.67 | 70±27.6 |
| MID4 | 4.5±2.20 | 0.81±0.399 | 3.3±1.60 | 3.3±1.57 | 54±26.1 |
| VRD1 | 4.7±1.66 | 0.72±0.285 | 3.2±1.37 | 3.0±1.36 | 50±22.1 |
| VRD2 | 5.0±2.19 | 0.73±0.329 | 3.2±1.33 | 3.2±1.32 | 52±22.0 |
| VRD3 | 4.7±1.22 | 0.71±0.204 | 3.3±0.85 | 3.3±0.83 | 53±13.8 |
| VRD4 | 1.6±0.88 | 0.24±0.155 | 1.1±0.61 | 1.1±0.61 | 18±10.2 |

^1^Values are presented as mean ± standard deviation (n = 4).

**Table S4**

Content of essential macro (g kg^-1^ dry matter, DM) and trace (mg kg^-1^ DM) elements in the studied *Lupinus* species for the four sowing dates (D) of the two locations (MI, Mirandela; VR, Vila Real)^1^.

|  | Ca | P | K | Mg | Na | Mn | Fe | Co | Cu | Zn | Mo |
| --- | --- | --- | --- | --- | --- | --- | --- | --- | --- | --- | --- |
| ***Lupinus albus* cv. Estoril** |  |  |  |  |  |  |  |  |  |  |  |
| MID1 | 5.7±0.56 | 2.1±0.25 | 10±1.1 | 1.9±0.14 | 1.3±1.07 | 3361±504.1 | 138±19.5 | 0.79±0.125 | 5.4±0.64 | 22±1.9 | 0.98±0.244 |
| MID2 | 5.8±0.83 | 2.4±0.24 | 11±0.9 | 2.0±0.21 | 1.1±0.74 | 3151±952.8 | 148±28.8 | 1.0±0.55 | 6.2±0.94 | 31±11.3 | 0.80±0.517 |
| MID3 | 5.2±0.66 | 2.7±0.32 | 12±1.6 | 2.0±0.15 | 1.2±0.60 | 1722±692.7 | 85±10.5 | 0.70±0.235 | 6.5±0.50 | 26±1.7 | 0.61±0.162 |
| MID4 | 5.6±0.79 | 2.6±0.28 | 13±1.4 | 2.1±0.06 | 1.1±0.27 | 2613±598.4 | 121±24.9 | 0.69±0.146 | 6.9±0.58 | 28±2.9 | 0.61±0.066 |
| VRD1 | 6.1±0.67 | 1.5±0.16 | 13±1.8 | 1.7±0.23 | 1.2±0.86 | 1384±791.3 | 126±23.8 | 2.1±0.71 | 11±1.6 | 34±11.1 | 0.25±0.059 |
| VRD2 | 6.3±0.93 | 1.6±0.20 | 12±0.7 | 1.6±0.22 | 1.1±0.12 | 1854±603.7 | 132±37.0 | 2.8±1.17 | 12±1.2 | 30±5.1 | 0.22±0.063 |
| VRD3 | 7.3±1.88 | 1.4±0.09 | 11±1.7 | 1.8±0.53 | 0.91±0.280 | 1577±958.2 | 148±33.7 | 2.7±0.49 | 12±2.1 | 30±5.0 | 0.23±0.117 |
| VRD4 | 6.4±0.56 | 1.6±0.14 | 11±1.5 | 1.7±0.13 | 0.75±0.250 | 1895±380.9 | 175±54.1 | 2.1±0.49 | 14±1.9 | 31±6.5 | 0.23±0.043 |
| ***Lupinus angustifolius* cv. Tango** |  |  |  |  |  |  |  |  |  |  |  |
| MID1 | 10±2.0 | 3.8±0.29 | 19±1.1 | 3.2±0.46 | 1.2±0.43 | 384±157.5 | 173±24.5 | 0.62±0.339 | 7.0±0.35 | 46±10.6 | 0.81±0.310 |
| MID2 | 10±2.8 | 3.2±0.64 | 19±3.4 | 3.2±0.56 | 1.2±0.41 | 275±90.2 | 206±109.7 | 0.51±0.190 | 8.0±1.85 | 61±22.4 | 0.66±0.295 |
| MID3 | 8.4±1.00 | 3.4±0.17 | 18±1.1 | 2.8±0.15 | 1.8±0.75 | 210±84.4 | 119±19.1 | 0.53±0.283 | 7.4±0.50 | 41±4.7 | 0.54±0.082 |
| MID4 | 7.6±1.01 | 3.8±0.27 | 20±2.3 | 2.6±0.29 | 1.6±0.57 | 144±31.3 | 93±7.9 | 0.38±0.145 | 7.4±0.49 | 43±11.4 | 0.62±0.121 |
| VRD1 | 12±2.5 | 1.5±0.19 | 12±0.6 | 3.2±0.29 | 1.3±0.45 | 499±232.2 | 308±97.9 | 3.6±1.49 | 11±0.9 | 84±43.5 | 0.44±0.174 |
| VRD2 | 12±1.5 | 1.6±0.11 | 13±1.6 | 3.0±0.38 | 1.4±0.13 | 438±175.8 | 242±36.0 | 3.4±1.36 | 11±1.0 | 58±18.1 | 0.40±0.176 |
| VRD3 | 11±4.2 | 1.6±0.24 | 12±2.2 | 2.6±0.79 | 1.1±0.33 | 804±590.3 | 270±106.2 | 3.9±1.93 | 12±1.0 | 46±9.0 | 0.32±0.140 |
| VRD4 | 12±2.1 | 1.6±0.08 | 14±1.9 | 2.9±0.39 | 1.1±0.23 | 455±118.5 | 350±70.7 | 3.2±0.92 | 11±1.5 | 101±71.1 | 0.44±0.153 |
| ***Lupinus luteus* cv. Cardiga** |  |  |  |  |  |  |  |  |  |  |  |
| MID1 | 7.3±0.65 | 3.6±0.79 | 26±3.1 | 3.1±0.09 | 1.8±0.36 | 414±153.7 | 193±36.0 | 0.50±0.110 | 9.0±1.17 | 74±19.1 | 0.82±0.232 |
| MID2 | 8.7±3.32 | 3.4±0.85 | 24±5.4 | 3.1±0.34 | 1.6±0.94 | 394±91.5 | 197±34.0 | 0.52±0.046 | 9.7±1.18 | 62±5.4 | 0.63±0.207 |
| MID3 | 6.8±0.90 | 3.7±0.34 | 22±3.2 | 2.7±0.21 | 1.0±0.19 | 312±40.7 | 124±14.9 | 0.38±0.018 | 8.9±0.82 | 57±5.9 | 0.83±0.122 |
| MID4 | 6.9±0.58 | 3.4±0.55 | 22±2.2 | 2.9±0.22 | 1.5±0.44 | 300±45.8 | 139±55.9 | 0.42±0.090 | 10±1.3 | 59±7.1 | 0.67±0.115 |
| VRD1 | 6.1±0.38 | 1.6±0.08 | 17±2.5 | 1.8±0.33 | 1.4±0.33 | 593±496.5 | 145±25.3 | 1.7±0.24 | 10±1.4 | 64±39.7 | 0.32±0.124 |
| VRD2 | 6.1±0.92 | 1.7±0.20 | 19±0.7 | 2.0±0.07 | 1.7±0.34 | 295±55.9 | 164±34.5 | 1.9±0.46 | 9.0±0.82 | 48±3.1 | 0.28±0.045 |
| VRD3 | 5.9±0.78 | 1.4±0.08 | 16±1.3 | 1.9±0.15 | 1.2±0.27 | 323±99.7 | 158±28.7 | 2.0±0.55 | 8.9±0.26 | 52±4.3 | 0.49±0.149 |
| VRD4 | 6.1±0.55 | 1.5±0.06 | 19±4.7 | 2.0±0.09 | 1.2±0.33 | 287±62.8 | 162±30.2 | 1.9±0.28 | 9.6±0.17 | 53±13.0 | 0.39±0.069 |
| **MTL**^2^ |  |  |  |  |  |  |  |  |  |  |  |
| Cattle | 15 | 7 | 20 | 6 | 45 (NaCl) | 2000 | 500 | 25 | 40 | 500 | 5 |
| Sheep | 15 | 6 | 20 | 6 | 40 (NaCl) | 2000 | 500 | 25 | 15 | 300 | 5 |

^1^Values are presented as mean ± standard deviation (n = 4).

^2^MTL, maximum tolerable level in the feed based on indexes of animal health (NRC, 2005).

**Table S5**

Content of toxic elements (mg kg^-1^ dry matter) in the studied *Lupinus* species for the four sowing dates (D) of the two locations (MI, Mirandela; VR, Vila Real)^1^.

|  | Cr | Li | Ti | Ni | Cs | Ba | Tl | Be | Sb |
| --- | --- | --- | --- | --- | --- | --- | --- | --- | --- |
| ***Lupinus albus* cv. Estoril** |  |  |  |  |  |  |  |  |  |
| MID1 | 0.24±0.058 | 0.15±0.032 | 4.09±1.382 | 1.28±0.287 | 0.46±0.231 | 8.14±1.224 | 0.01±0.004 | 0.01±0.001 | 0.004±0.0013 |
| MID2 | 0.22±0.081 | 0.19±0.014 | 4.29±0.545 | 1.54±0.342 | 0.44±0.282 | 9.31±2.117 | 0.01±0.005 | 0.01±0.002 | 0.004±0.0007 |
| MID3 | 0.13^2^ | 0.15±0.036 | 2.44±0.379 | 1.50±0.359 | 0.33±0.059 | 8.42±0.849 | 0.01±0.001 | - | - |
| MID4 | 0.21±0.075 | 0.17±0.036 | 3.82±0.914 | 1.58±0.316 | 0.49±0.104 | 8.49±1.529 | 0.01±0.001 | 0.01±0.001 | 0.003±0.0007 |
| VRD1 | 0.33±0.195 | 0.24±0.043 | 2.88±1.241 | 1.30±0.432 | 2.27±0.558 | 25.1±4.11 | 0.10±0.099 | 0.03±0.015 | 0.003±0.0010 |
| VRD2 | 0.22±0.081 | 0.27±0.093 | 2.84±1.110 | 1.34±0.336 | 2.58±0.641 | 22.6±3.63 | 0.05±0.009 | 0.04±0.017 | 0.004±0.0014 |
| VRD3 | 0.19±0.031 | 0.29±0.007 | 3.55±1.328 | 1.24±0.253 | 1.92±0.549 | 33.3±13.87 | 0.06±0.032 | 0.04±0.006 | 0.004±0.0007 |
| VRD4 | 0.26±0.103 | 0.32±0.075 | 4.08±1.479 | 1.40±0.356 | 2.48±0.705 | 30.0±4.86 | 0.04±0.009 | 0.05±0.018 | 0.004±0.0017 |
| ***Lupinus angustifolius* cv. Tango** |  |  |  |  |  |  |  |  |  |
| MID1 | 0.43±0.086 | 0.27±0.058 | 8.86±1.323 | 1.39±0.188 | 0.27±0.057 | 24.8±4.18 | 0.01±0.004 | 0.02±0.004 | 0.004±0.0009 |
| MID2 | 0.68±0.590 | 0.30±0.102 | 12.4±9.75 | 1.60±0.435 | 0.26±0.051 | 22.2±8.58 | 0.01±0.007 | 0.02±0.004 | 0.007±0.0043 |
| MID3 | 0.34±0.047 | 0.30±0.076 | 5.78±1.880 | 1.55±0.173 | 0.28±0.042 | 27.2±5.37 | 0.01±0.004 | 0.01±0.003 | 0.003±0.0004 |
| MID4 | 0.19±0.053 | 0.22±0.024 | 3.90±0.830 | 1.35±0.138 | 0.28±0.067 | 26.1±7.56 | 0.01±0.003 | 0.01±0.001 | 0.004^2^ |
| VRD1 | 0.58±0.159 | 0.46±0.094 | 9.11±1.834 | 1.52±0.290 | 0.81±0.185 | 94.9±33.81 | 0.15±0.056 | 0.09±0.034 | 0.009±0.0025 |
| VRD2 | 0.50±0.247 | 0.36±0.079 | 8.12±3.145 | 1.32±0.193 | 0.73±0.146 | 77.2±20.74 | 0.13±0.036 | 0.06±0.016 | 0.007±0.0016 |
| VRD3 | 0.45±0.209 | 0.47±0.167 | 9.22±5.423 | 1.45±0.270 | 1.38±0.855 | 66.1±29.71 | 0.08±0.037 | 0.06±0.024 | 0.007±0.0030 |
| VRD4 | 0.58±0.044 | 0.56±0.110 | 12.6±4.246 | 1.64±0.396 | 0.90±0.110 | 84.4±19.30 | 0.14±0.031 | 0.07±0.014 | 0.009±0.0037 |
| ***Lupinus luteus* cv. Cardiga** |  |  |  |  |  |  |  |  |  |
| MID1 | 0.27±0.096 | 0.23±0.071 | 9.15±4.652 | 1.33±0.265 | 0.32±0.090 | 12.8±2.76 | 0.03±0.008 | 0.01±0.004 | 0.007±0.0039 |
| MID2 | 0.27±0.089 | 0.26±0.068 | 10.9±2.516 | 1.48±0.229 | 0.30±0.035 | 17.7±11.72 | 0.02±0.012 | 0.01±0.008 | 0.007±0.0018 |
| MID3 | 0.14±0.015 | 0.17±0.027 | 5.10±0.869 | 1.53±0.040 | 0.25±0.047 | 9.94±0.386 | 0.02±0.003 | 0.01±0.001 | 0.005±0.0017 |
| MID4 | 0.24±0.172 | 0.19±0.040 | 7.13±5.601 | 1.70±0.148 | 0.32±0.066 | 11.3±1.40 | 0.03±0.010 | 0.01±0.002 | 0.004±0.0020 |
| VRD1 | 0.24±0.025 | 0.24±0.035 | 4.59±1.325 | 1.08±0.060 | 1.98±0.570 | 24.3±2.68 | 0.11±0.041 | 0.03±0.005 | 0.005±0.0011 |
| VRD2 | 0.28±0.080 | 0.24±0.068 | 5.65±1.726 | 1.11±0.168 | 1.72±0.426 | 25.0±2.58 | 0.17±0.018 | 0.02±0.006 | 0.005±0.0012 |
| VRD3 | 0.29±0.081 | 0.29±0.043 | 6.10±0.949 | 1.06±0.120 | 2.07±0.373 | 25.4±4.53 | 0.14±0.008 | 0.02±0.008 | 0.004±0.0005 |
| VRD4 | 0.21±0.064 | 0.26±0.072 | 4.35±0.965 | 1.09±0.078 | 1.89±1.000 | 33.7±10.78 | 0.18±0.026 | 0.02±0.004 | 0.005±0.0011 |

^1^Values are presented as mean ± standard deviation (n = 4).

^2^Only detected in one plot.

**Table S5 (conclusion)**

Content of toxic elements (mg kg^-1^ dry matter) in the studied *Lupinus* species for the four sowing dates (D) of the two locations (MI, Mirandela; VR, Vila Real)^1^.

|  | Rb | Sr | Cd | Pb | Hg |
| --- | --- | --- | --- | --- | --- |
| ***Lupinus albus* cv. Estoril** |  |  |  |  |  |
| MID1 | 30±10.0 | 22±2.5 | 0.03±0.011 | 0.54±0.836 | - |
| MID2 | 29±16.1 | 21±1.1 | 0.03±0.008 | 0.14±0.060 | - |
| MID3 | 27±5.2 | 21±3.1 | 0.02±0.010 | 0.10±0.027 | - |
| MID4 | 35±5.9 | 20±3.6 | 0.02±0.006 | 0.13±0.027 | - |
| VRD1 | 89±14.8 | 18±0.9 | 0.05±0.039 | 0.75±0.048 | 0.03±0.009 |
| VRD2 | 88±18.9 | 16±2.0 | 0.02±0.009 | 0.71±0.185 | 0.06±0.011 |
| VRD3 | 79±7.4 | 20±8.4 | 0.03±0.026 | 0.82±0.205 | 0.03±0.008 |
| VRD4 | 85±5.6 | 17±1.4 | 0.02±0.003 | 0.78±0.147 | 0.04±0.015 |
| ***Lupinus angustifolius* cv. Tango** |  |  |  |  |  |
| MID1 | 39±4.0 | 42±6.8 | 0.05±0.016 | 0.15±0.033 | - |
| MID2 | 38±6.4 | 39±12.3 | 0.08±0.060 | 0.24±0.117 | - |
| MID3 | 40±6.5 | 41±5.8 | 0.05±0.016 | 0.13±0.020 | - |
| MID4 | 40±10.1 | 36±5.6 | 0.03±0.001 | 0.09±0.026 | - |
| VRD1 | 70±10.7 | 44±8.7 | 0.10±0.022 | 1.38±0.272 | 0.03±0.006 |
| VRD2 | 69±13.2 | 38±4.2 | 0.10±0.029 | 1.38±0.357 | 0.03±0.011 |
| VRD3 | 78±8.7 | 36±16.5 | 0.08±0.044 | 1.33±0.481 | 0.03±0.007 |
| VRD4 | 76±4.8 | 39±7.2 | 0.10±0.027 | 1.40±0.331 | 0.03±0.008 |
| ***Lupinus luteus* cv. Cardiga** |  |  |  |  |  |
| MID1 | 50±13.8 | 27±4.7 | 0.14±0.024 | 0.21±0.025 | - |
| MID2 | 49±5.7 | 34±15.9 | 0.11±0.044 | 0.20±0.041 | - |
| MID3 | 41±4.3 | 24±2.1 | 0.13±0.019 | 0.11±0.007 | - |
| MID4 | 49±7.8 | 26±1.5 | 0.15±0.023 | 0.16±0.068 | - |
| VRD1 | 110±25.8 | 18±2.9 | 0.12±0.072 | 0.95±0.325 | 0.02±0.030 |
| VRD2 | 107±16.5 | 17±1.6 | 0.14±0.035 | 0.78±0.134 | - |
| VRD3 | 114±11.3 | 16±3.1 | 0.11±0.017 | 0.67±0.172 | 0.03±0.002 |
| VRD4 | 104±15.5 | 18±0.1 | 0.14±0.024 | 0.75±0.075 | 0.03±0.009 |

^1^Values are presented as mean ± standard deviation (n = 4).

**Table S6**

Content (mg kg^-1^ dry matter) of indole, piperidine, quinolizidine (bicyclic and tetracyclic), and total alkaloids of the studied *Lupinus* species for the four sowing dates (D) of the two locations (MI, Mirandela; VR, Vila Real). -, not detected; < (value), below the limit of quantification^1^.

|  | Indole | Piperidine | Quinolizidine | | Total |
| --- | --- | --- | --- | --- | --- |
|  |  |  | Bicyclic | Tetracyclic |  |
| ***Lupinus albus* cv. Estoril** |  |  |  |  |  |
| MID1 | - | 43.4 ± 8.53 | - | 89.1 ± 15.25 | 133 ± 12.5 |
| MID2 | - | 33.6 ± 4.71 | - | 89.7 ± 16.15 | 123 ± 16.0 |
| MID3 | - | 40.1 ± 7.79 | - | 88.4 ± 7.26 | 128 ± 13.5 |
| MID4 | - | 45.7 ± 10.40 | - | 98.0 ± 21.40 | 144 ± 28.7 |
| VRD1 | - | 25.0 ± 6.46 | - | 41.3 ± 1.90 | 66.3 ± 6.31 |
| VRD2 | - | 29.0 ± 5.84 | - | 55.9 ± 7.94 | 84.9 ± 8.70 |
| VRD3 | - | 25.1 ± 12.93 | - | 46.0 ± 20.72 | 71.1 ± 33.62 |
| VRD4 | - | 32.1 ± 6.79 | - | 55.0 ± 16.07 | 87.1 ± 22.1 |
| ***Lupinus angustifolius* cv. Tango** |  |  |  |  |  |
| MID1 | - | - | - | 27.0 ± 18.21 | 27.7 ± 18.22 |
| MID2 | - | < 2.57 | - | 32.2 ± 19.72 | 33.6 ± 20.49 |
| MID3 | - | < 2.57 | - | 15.6 ± 3.84 | 16.7 ± 3.12 |
| MID4 | - | - | - | 17.5 ± 9.94 | 18.2 ± 9.94 |
| VRD1 | - | - | - | 3.49 ± 1.552 | 4.19 ± 1.552 |
| VRD2 | - | - | - | 3.03 ± 1.068 | 3.73 ± 1.068 |
| VRD3 | - | 4.95 ± 4.227 | - | 8.89 ± 2.464 | 13.8 ± 5.78 |
| VRD4 | - | 13.0 ± 14.45 | - | 10.7 ± 9.30 | 23.7 ± 23.73 |
| ***Lupinus luteus* cv. Cardiga** |  |  |  |  |  |
| MID1 | 341 ± 46.0 | 488 ± 277.5 | 2086 ± 480.4 | 198 ± 44.8 | 3114 ± 580.7 |
| MID2 | 425 ± 74.2 | 131 ± 111.6 | 2322 ± 383.4 | 241 ± 48.3 | 3119 ± 422.9 |
| MID3 | 474 ± 47.4 | 110 ± 16.7 | 1789 ± 131.1 | 235 ± 18.9 | 2608 ± 139.6 |
| MID4 | 480 ± 34.7 | 138 ± 61.5 | 1383 ± 920.5 | 244 ± 19.5 | 2245 ± 930.1 |
| VRD1 | 269 ± 70.5 | 260 ± 96.4 | 2439 ± 457.8 | 284 ± 46.8 | 3252 ± 519.1 |
| VRD2 | 261 ± 6.6 | 238 ± 97.2 | 2056 ± 293.1 | 242 ± 43.0 | 2797 ± 405.3 |
| VRD3 | 296 ± 63.0 | 269 ± 58.6 | 2101 ± 69.3 | 325 ± 28.6 | 2991 ± 36.0 |
| VRD4 | 288 ± 35.7 | 269 ± 84.6 | 2137 ± 129.1 | 275 ± 12.3 | 2971 ± 169.8 |

^1^Values are presented as mean ± standard deviation (n = 4).

**Table S7**

Content (mg kg^-1^ dry matter) of individual indole, piperidine, and bicyclic quinolizidine alkaloids of the studied *Lupinus* species for the four sowing dates (D) of the two locations (MI, Mirandela; VR, Vila Real). -, not detected; <(value), below the limit of quantification^1^.

|  | Indole | |  | Piperidine | |  | Bicyclic quinolizidine | | |
| --- | --- | --- | --- | --- | --- | --- | --- | --- | --- |
|  | Gramine | Gramine derivative |  | Smipine | Ammodendrine |  | Lupinine | Lusitanine | Feruloyllupinine |
| ***Lupinus albus* cv. Estoril** |  |  |  |  |  |  |  |  |  |
| MID1 | - | - |  | 27.2 ± 6.2 | 16.2 ± 3.9 |  | - | - | - |
| MID2 | - | - |  | 22.8 ± 5.2 | 10.8 ± 3.2 |  | - | - | - |
| MID3 | - | - |  | 31.3 ± 5.1 | 8.75 ± 3.09 |  | - | - | - |
| MID4 | - | - |  | 36.8 ± 7.9 | 8.88 ± 2.67 |  | - | - | - |
| VRD1 | - | - |  | 17.1 ± 5.2 | 7.88 ± 1.25 |  | - | - | - |
| VRD2 | - | - |  | 18.6 ± 5.9 | 10.3 ± 1.7 |  | - | - | - |
| VRD3 | - | - |  | 16.5 ± 9.8 | 8.62 ± 3.38 |  | - | - | - |
| VRD4 | - | - |  | 22.3 ± 4.7 | 9.89 ± 2.19 |  | - | - | - |
| ***Lupinus angustifolius* cv. Tango** |  |  |  |  |  |  |  |  |  |
| MID1 | - | - |  | - | - |  | - | - | - |
| MID2 | - | - |  | - | < 2.57 |  | - | - | - |
| MID3 | - | - |  | - | < 2.57 |  | - | - | - |
| MID4 | - | - |  | - | - |  | - | - | - |
| VRD1 | - | - |  | - | - |  | - | - | - |
| VRD2 | - | - |  | - | - |  | - | - | - |
| VRD3 | - | - |  | - | 4.95 ± 4.23 |  | - | - | - |
| VRD4 | - | - |  | - | 13.0 ± 14.5 |  | - | - | - |
| ***Lupinus luteus* cv. Cardiga** |  |  |  |  |  |  |  |  |  |
| MID1 | 327 ± 45 | 19.7 ± 4.8 |  | - | 488 ± 278 |  | 1997 ± 467 | 82.4 ± 15.3 | 13.9 ± 10.5 |
| MID2 | 386 ± 56 | 38.8 ± 18.3 |  | - | 131 ± 112 |  | 2239 ± 357 | 78.9 ± 27.5 | < 12.71 |
| MID3 | 447 ± 42 | 27.5 ± 8.1 |  | - | 110 ± 17 |  | 1754 ± 125 | 32.4 ± 9.2 | - |
| MID4 | 440 ± 36 | 40.0 ± 14.5 |  | - | 138 ± 61 |  | 1789 ± 257 | 38.9 ± 3.5 | < 12.71 |
| VRD1 | 257 ± 75 | 16.9 ± 3.6 |  | - | 260 ± 96 |  | 2361 ± 448 | 67.1 ± 10.6 | 13.6 ± 11.5 |
| VRD2 | 243 ± 6.5 | 18.3 ± 7.3 |  | - | 238 ± 97 |  | 2008 ± 288 | 40.3 ± 9.5 | < 12.71 |
| VRD3 | 277 ± 58.6 | 18.7 ± 9.0 |  | - | 269 ± 59 |  | 2042 ± 58 | 35.7 ± 5.5 | 23.2 ± 23.1 |
| VRD4 | 264 ± 30.6 | 24.4 ± 5.8 |  | - | 269 ± 85 |  | 2081 ± 133 | 43.3 ± 5.8 | 17.7 ± 9.6 |

^1^Values are presented as mean ± standard deviation (n = 4).

**Table S8**

Content (mg kg^-1^ dry matter) of individual tetracyclic quinolizidine alkaloids of the studied *Lupinus* species for the four sowing dates (D) of the two locations (MI, Mirandela; VR, Vila Real). -, not detected; <(value), below the limit of quantification^1^.

|  | α-iso-sparteine | Sparteine | β-*iso*-sparteine | Dehydrosparteine | Hydroxy-β-isosparteine | Multiflorine | Lupanine | 7-hydroxylupanine | 13-α-angelolyoxylupanine |
| --- | --- | --- | --- | --- | --- | --- | --- | --- | --- |
| ***Lupinus albus* cv. Estoril** |  |  |  |  |  |  |  |  |  |
| MID1 | - | 27.2 ± 10.87 | - | - |  | 5.56 ± 1.952 | 37.5 ± 9.70 | 15.5 ± 4.73 | 3.40 ± 0.812 |
| MID2 | - | 18.5 ± 11.99 | - | - | - | 4.42 ± 1.623 | 45.1 ± 6.21 | 19.5 ± 11.20 | 2.82 ± 0.627 |
| MID3 | - | 6.75 ± 4.976 | - | - | - | < 2.57 | 38.3 ± 2.50 | 37.3 ± 2.88 | 3.90 ± 0.411 |
| MID4 | - | 26.5 ± 25.20 | - | - | - | 5.54 ± 3.472 | 40.6 ± 11.41 | 20.8 ± 4.82 | 4.61 ± 1.561 |
| VRD1 | - | 17.4 ± 3.57 | - | - | - | < 2.57 | 18.5 ± 1.74 | - | < 2.57 |
| VRD2 | - | 24.2 ± 4.43 | - | - | - | 3.50 ± 0.927 | 21.2 ± 2.47 | 5.58 ± 1.925 | < 2.57 |
| VRD3 | - | 13.8 ± 7.03 | - | - | - | < 2.57 | 22.6 ± 7.78 | 4.76 ± 3.639 | < 2.57 |
| VRD4 | - | 22.0 ± 7.33 | - | - | - | 3.79 ± 1.545 | 22.6 ± 5.41 | 4.54 ± 2.104 | < 2.57 |
| ***Lupinus angustifolius* cv. Tango** |  |  |  |  |  |  |  |  |  |
| MID1 | - | - | - | - | - | - | 26.3 ± 18.41 | - | - |
| MID2 | - | 4.45 ± 3.136 | - | - | - | - | 30.0 ± 18.59 | - | - |
| MID3 | - | - | - | - | - | - | 15.3 ± 4.44 | - | - |
| MID4 | - | < 2.11 | - | - | - | - | 16.9 ± 9.73 | - | - |
| VRD1 | - | - | - | - | - | - | 3.49 ± 1.552 | - | - |
| VRD2 | - | - | - | - | - | - | 3.03 ± 1.068 | - | - |
| VRD3 | - | 5.12 ± 2.596 | - | - | - | - | 3.77 ± 0.160 | - | - |
| VRD4 | - | 8.09 ± 9.885 | - | - | - | - | 2.59 ± 0.811 | - | - |
| ***Lupinus luteus* cv. Cardiga** |  |  |  |  |  |  |  |  |  |
| MID1 | 6.78 ± 1.922 | 172 ± 48.5 | 2.30 ± 0.747 | 5.43 ± 1.762 | 10.5 ± 6.88 | - | < 2.57 | - | - |
| MID2 | 7.88 ± 3.514 | 220 ± 43.3 | 7.70 ± 8.460 | 3.67 ± 1.177 | 3.95 ± 2.523 | - | < 2.57 | - | - |
| MID3 | 5.67 ± 0.155 | 217 ± 18.1 | 2.45 ± 0.207 | 2.32 ± 0.836 | 6.85 ± 1.919 | - | - | - | - |
| MID4 | 6.28 ± 0.978 | 225 ± 16.1 | 2.93 ± 0.270 | 2.28 ± 0.671 | 6.45 ± 2.905 | - | < 2.57 | - | - |
| VRD1 | 9.02 ± 4.926 | 259 ± 46.6 | 3.67 ± 0.914 | 4.20 ± 0.363 | 8.24 ± 2.353 | - | < 2.57 | - | - |
| VRD2 | 5.67 ± 0.598 | 221 ± 39.3 | 2.78 ± 0.971 | 3.98 ± 1.934 | 7.61 ± 0.851 | - | < 2.57 | - | - |
| VRD3 | 9.47 ± 1.831 | 292 ± 28.7 | 5.05 ± 0.425 | 6.41 ± 2.051 | 11.0 ± 3.51 | - | - | - | - |
| VRD4 | 7.17 ± 0.518 | 250 ± 8.5 | 3.93 ± 0.414 | 5.01 ± 1.494 | 8.65 ± 3.782 | - | < 2.57 | - | - |

^1^Values are presented as mean ± standard deviation (n = 4).

**
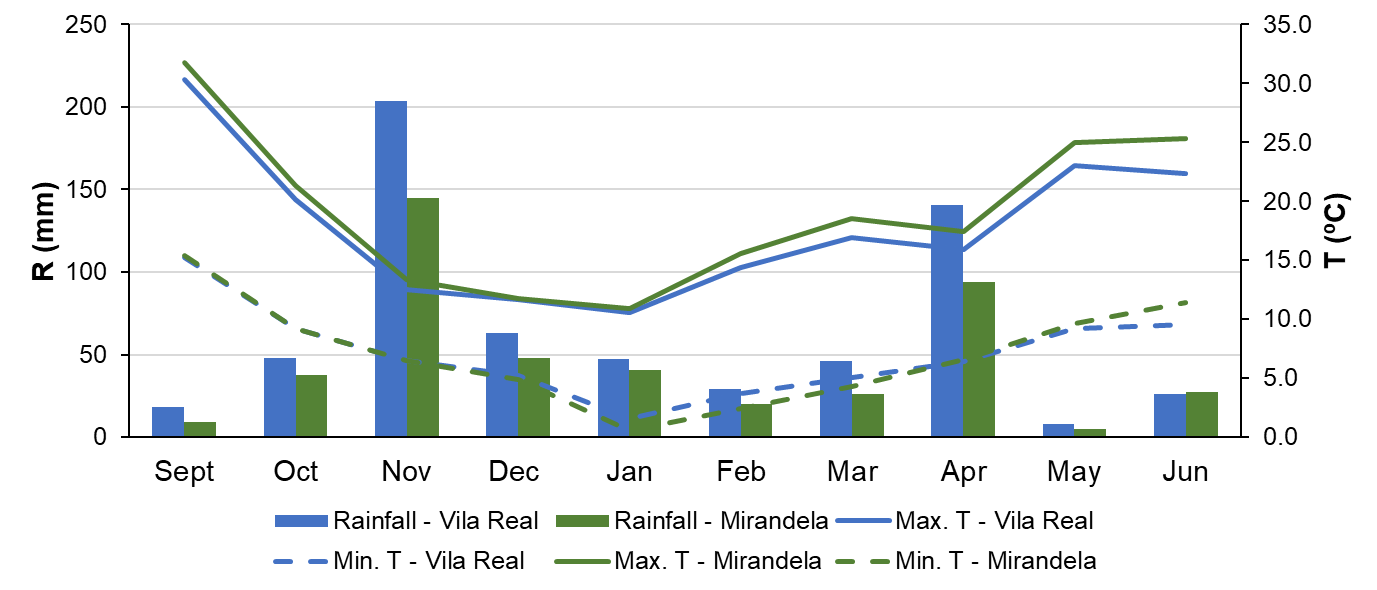
**

**Fig. S1.** Average maximum and minimum temperatures and total rainfall in Mirandela and Vila Real per month during the cultivation period. R, rainfall; Max. T, average maximum temperature; Min. T, average minimum temperature.

| 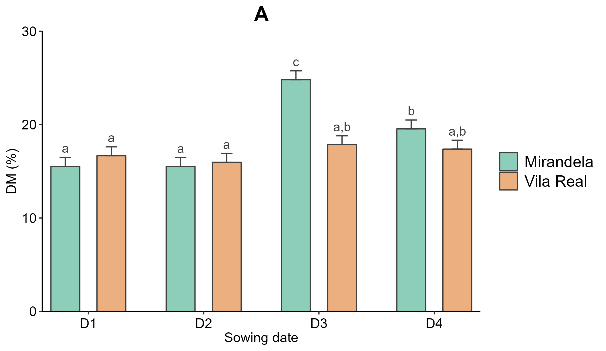 | 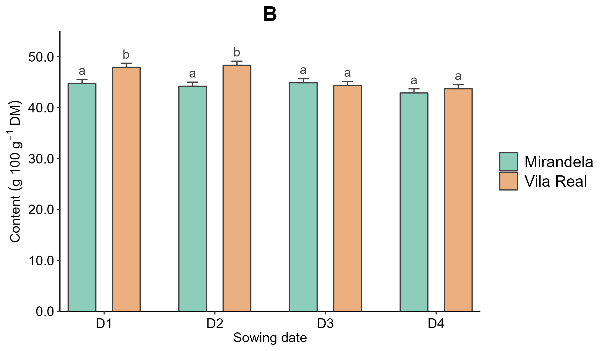 |
| --- | --- |
| 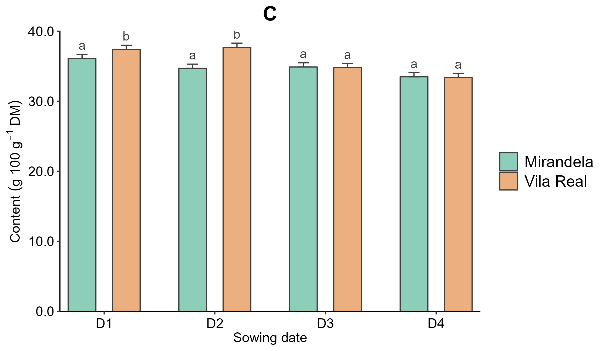 | 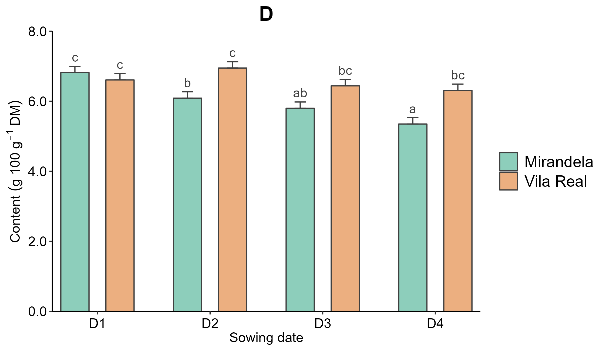 |
| 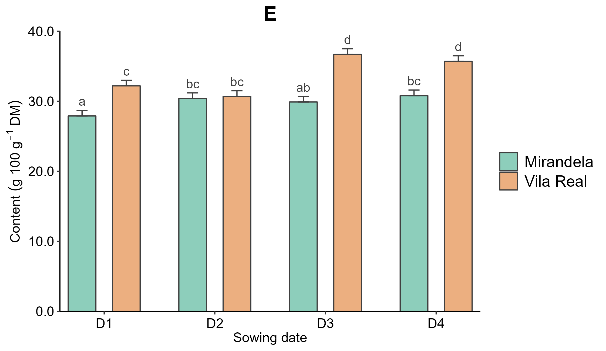 | 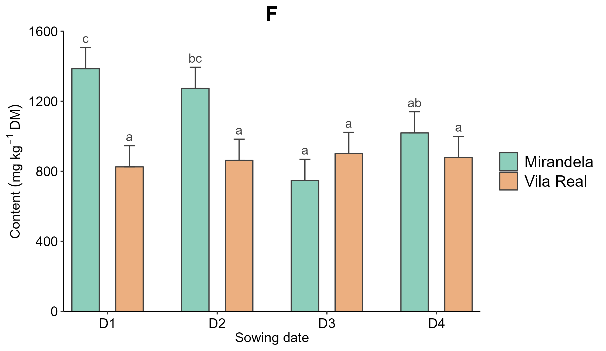 |
| 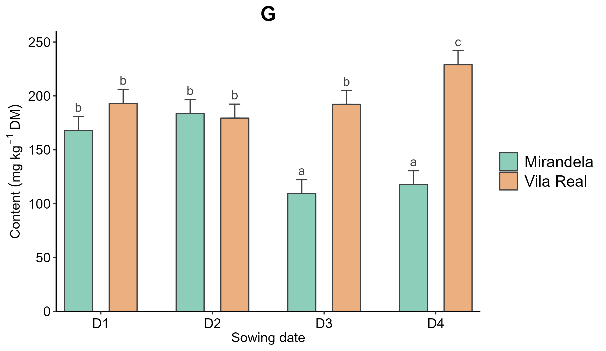 | 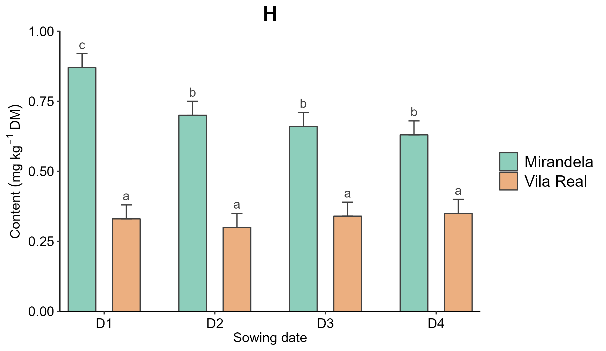 |
| 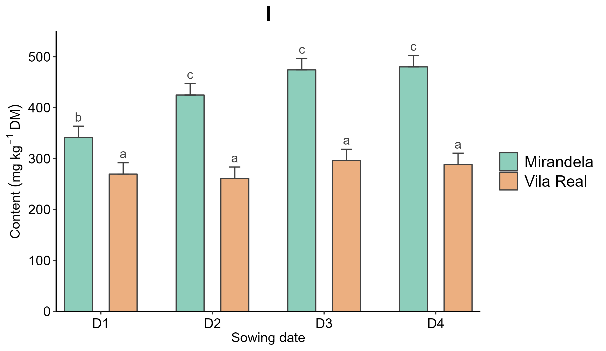 | 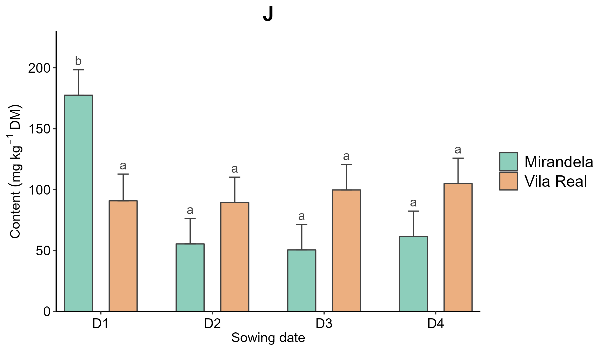 |
| 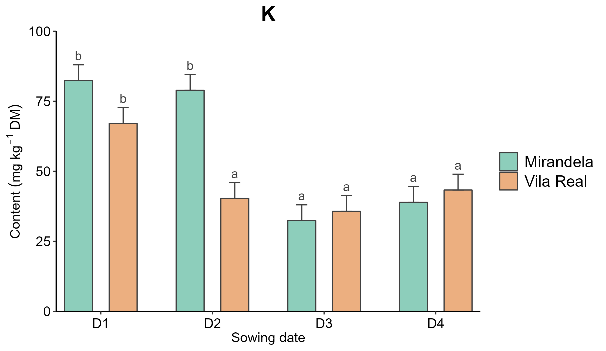 | 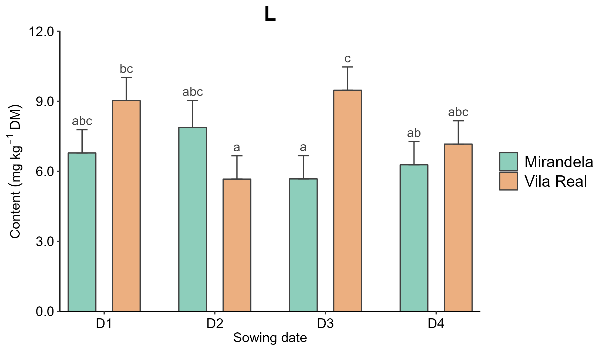 |
| 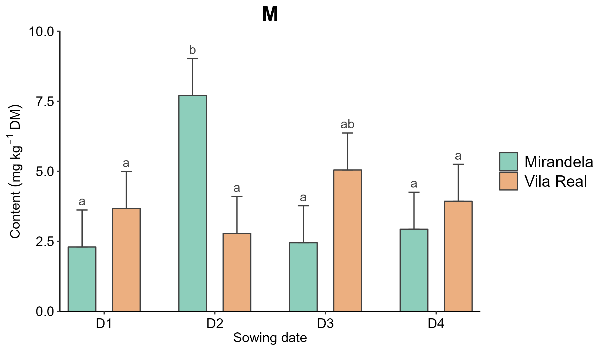 | 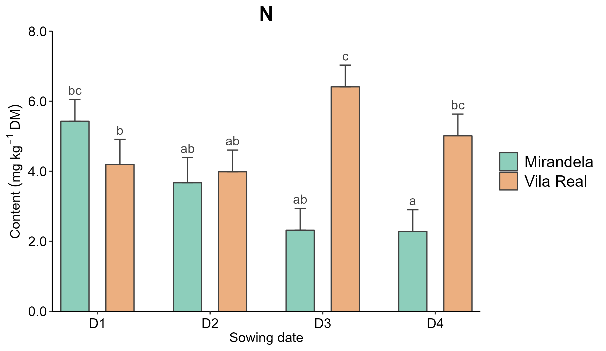 |
| 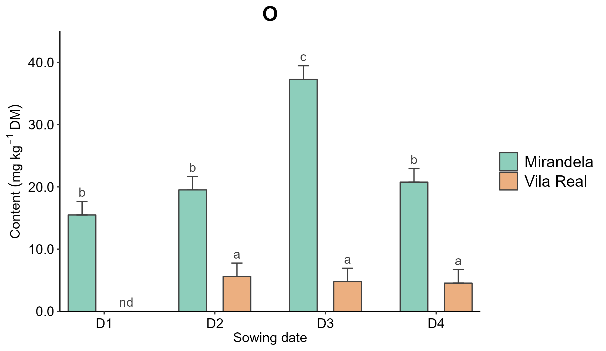 |  |

**Fig. S2.** Effect of interaction between sowing date and local on (A) dry matter, DM, (B) neutral detergent fibre, NDF, (C) acid detergent fibre, ADF, (D) acid detergent lignin, ADL, (E) non-structural carbohydrates, NSC, (F) manganese, Mn, (G) iron, Fe, (H) molybdenum, Mo, (I) indole alkaloids, (J) piperidine alkaloids, (K) lusitanine, (L) α-*iso*-sparteine, (M) β-*iso*-sparteine, (N) dehydrosparteine, (O) 7-hydroxylupanine in different *Lupinus* cultivars. ^a,b^means within each panel with different superscript letters are significantly different (*p* < 0.05).

| 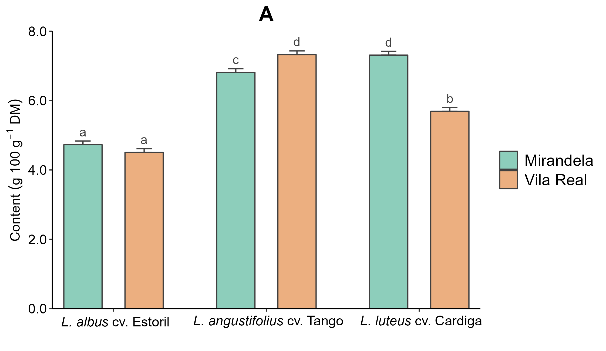 | 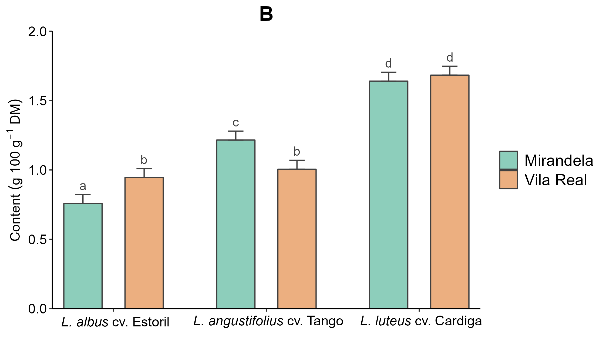 |
| --- | --- |
| 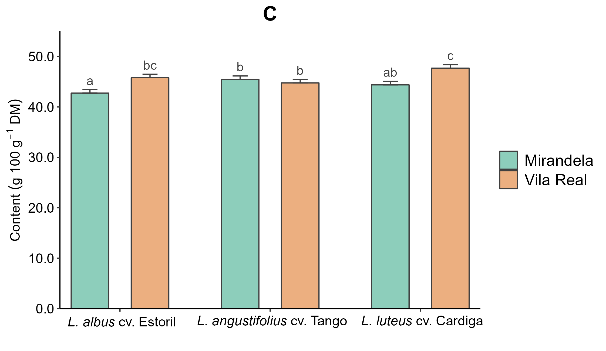 | 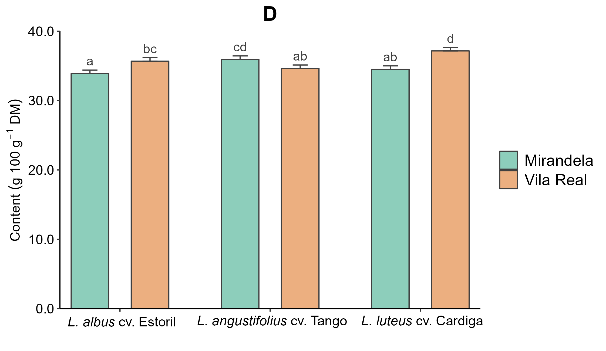 |
| 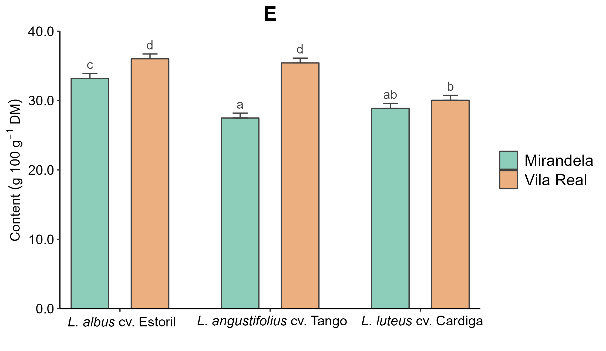 | 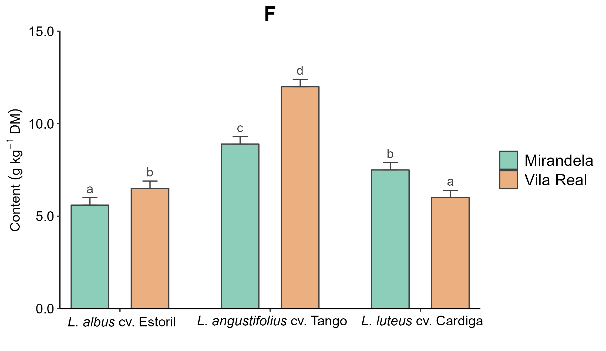 |
| 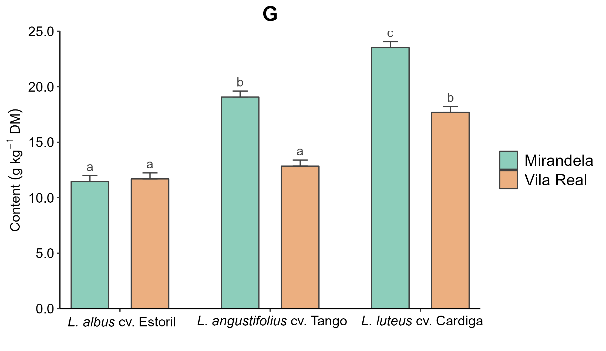 | 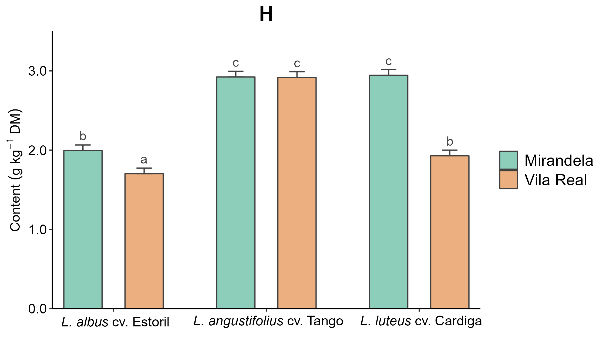 |
| 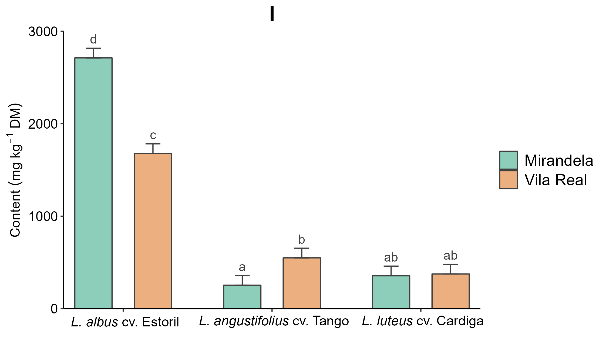 | 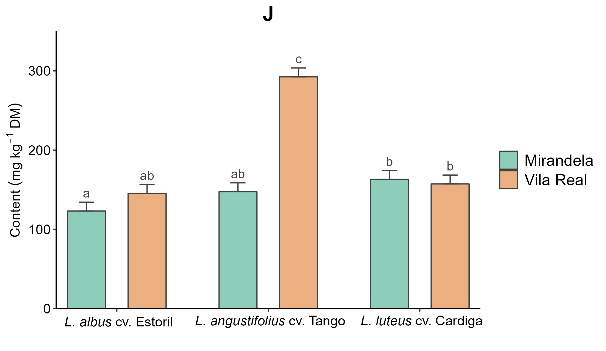 |
| 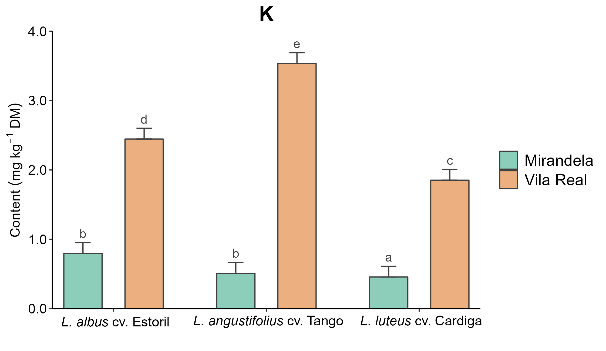 | 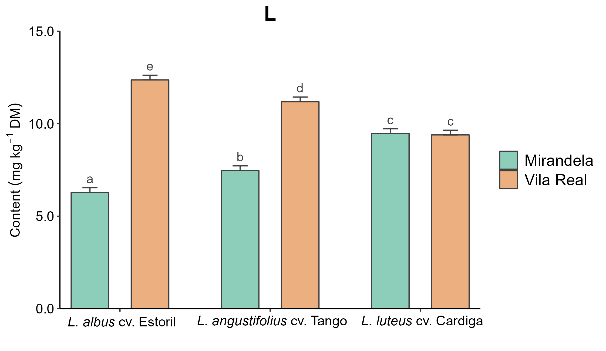 |
| 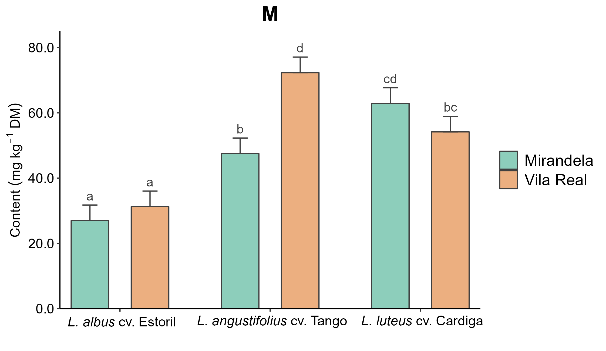 | 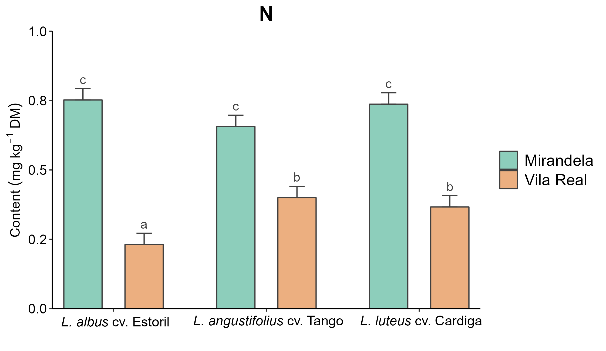 |
| 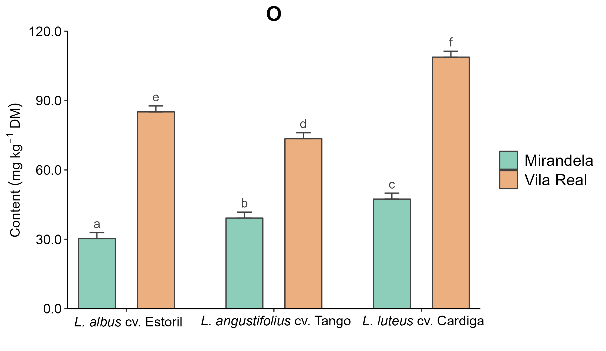 | 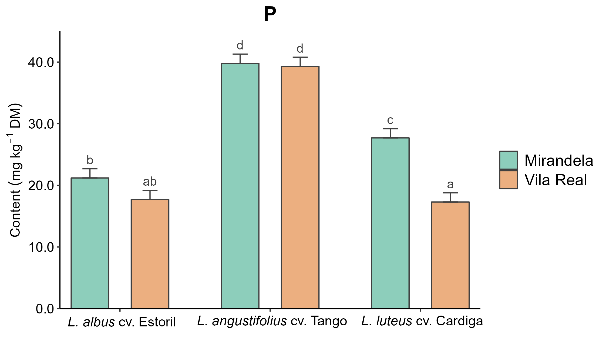 |
| 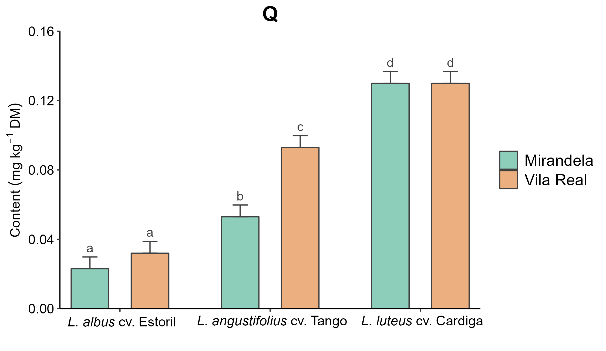 | 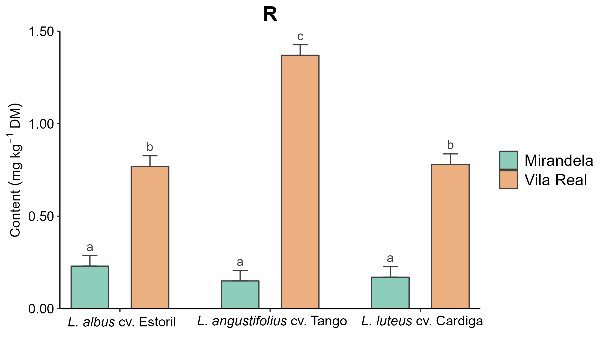 |
| 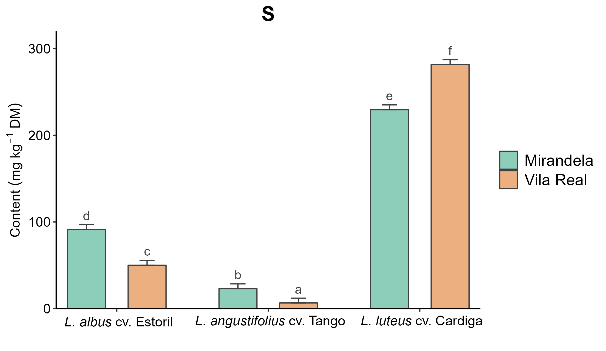 |  |

**Fig. S3.** Effect of interaction between species and location on (A) ash, (B) ether extract, EE, (C) neutral detergent fibre, NDF, (D) acid detergent fibre, ADF, (E) non-structural carbohydrates, NSC, (F) calcium, Ca, (G) potassium, K, (H) magnesium, Mg, (I) manganese, Mn, (J) iron, Fe, (K) cobalt, Co, (L) copper, Cu, (M) zinc, Zn, (N) molybdenum, Mo, (O) rubydium, Rb, (P) strontium, Sr, (Q) cadmium, Cd, (R) lead, Pb, (S) tetracyclic quinolizidine alkaloids in different *Lupinus* cultivars. ^a,b^means within each panel with different superscript letters are significantly different (*p* < 0.05).

| 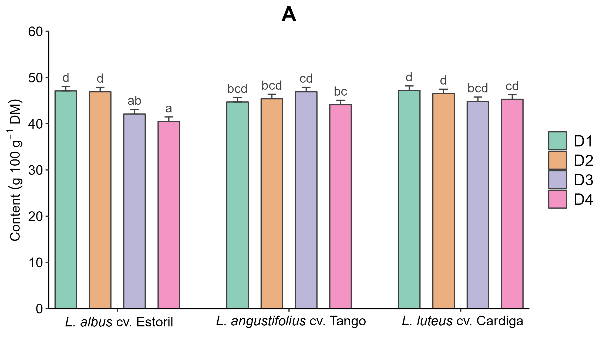 | 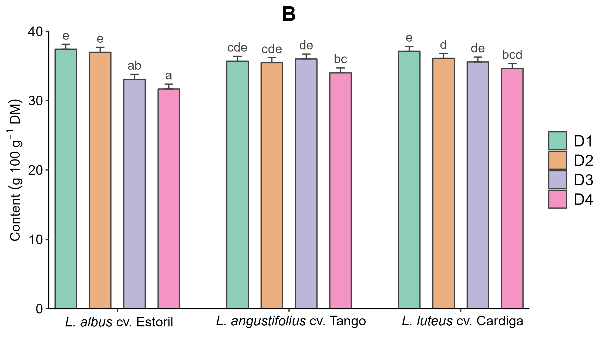 |
| --- | --- |

| 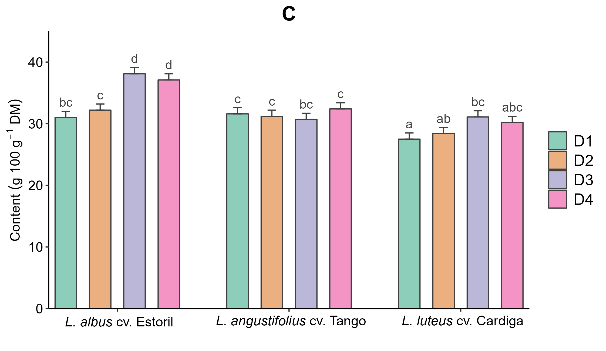 | 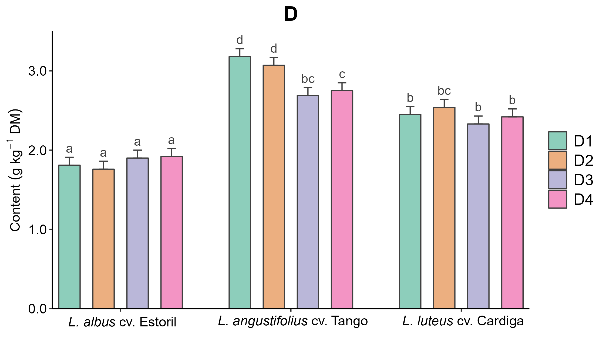 |
| --- | --- |

| 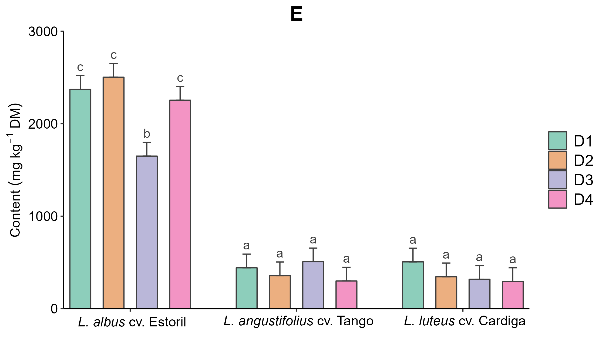 | 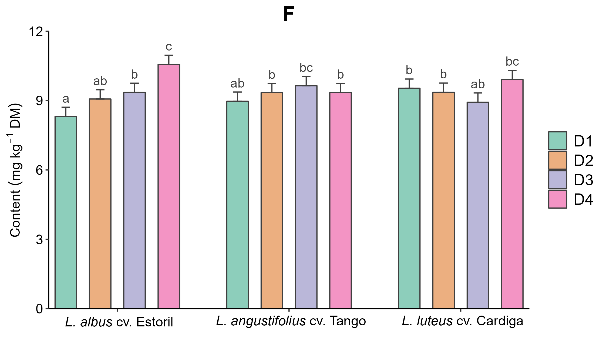 |
| --- | --- |

| 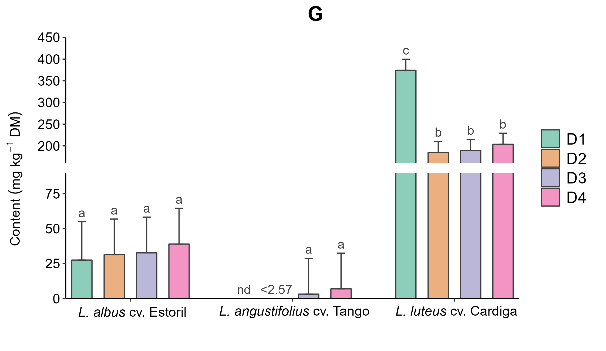 | 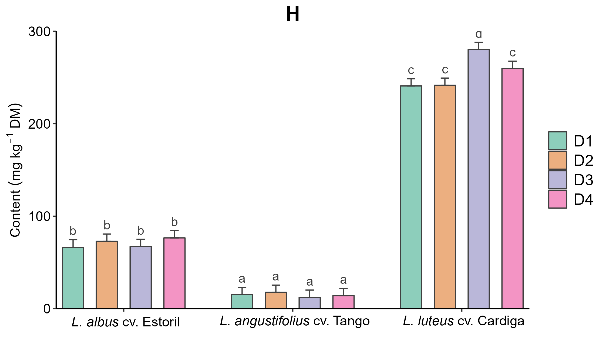 |
| --- | --- |

**Fig. S4.** Effect of interaction between species and sowing date on (A) neutral detergent fibre, NDF, (B) acid detergent fibre, ADF, (C) non-structural carbohydrates, NSC, (D) magnesium, Mg, (E) manganese, Mn, (F) Copper, Cu, (G) piperidine alkaloids, and (H) tetracyclic quinolizidine alkaloids contents in different *Lupinus* species. ^a,b^means within each panel with different superscript letters are significantly different (*p* < 0.05). nd means not detected. <(value) means lower than the limit of quantification.
